# Giant Periodic Pseudomagnetic Fields in Strained Kagome Magnet FeSn Epitaxial Films on SrTiO_3_(111) Substrate

**DOI:** 10.1021/acs.nanolett.3c00345

**Published:** 2023-03-13

**Authors:** Huimin Zhang, Michael Weinert, Lian Li

**Affiliations:** † Department of Physics and Astronomy, West Virginia University, Morgantown, West Virginia 26506, United States; ‡ State Key Laboratory of Structural Analysis, Optimization and CAE Software for Industrial Equipment, Dalian University of Technology, Dalian, 116024, China; § Department of Physics, University of Wisconsin, Milwaukee, Wisconsin 53201, United States

**Keywords:** periodic pseudomagnetic fields, kagome magnet, FeSn, strain engineering, molecular beam epitaxy, scanning tunneling microscopy/spectroscopy

## Abstract

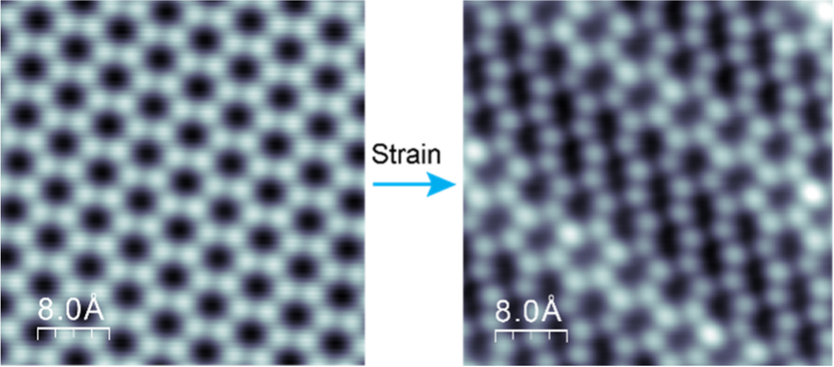

Quantum materials, particularly Dirac materials with linearly dispersing bands, can be effectively tuned by strain-induced lattice distortions leading to a pseudomagnetic field that strongly modulates their electronic properties. Here, we grow kagome magnet FeSn films, consisting of alternatingly stacked Sn_2_ honeycomb (stanene) and Fe_3_Sn kagome layers, on SrTiO_3_(111) substrates by molecular beam epitaxy. Using scanning tunneling microscopy/spectroscopy, we show that the Sn honeycomb layer can be periodically deformed by epitaxial strain for a film thickness below 10 nm, resulting in differential conductance peaks consistent with Landau levels generated by a pseudomagnetic field greater than 1000 T. Our findings demonstrate the feasibility of strain engineering the electronic properties of topological magnets at the nanoscale.

Dirac materials are characterized by linearly dispersive energy bands, hosting massless Dirac Fermions.^1^ When subjected to strain, which introduces position-dependent perturbations, electronic properties can be significantly impacted.^2^ For graphene, a prototypical Dirac material, structural distortions due to strain can modify the hopping energies between π orbitals in different sublattices, which causes the Dirac points to shift to opposite directions analogous to an applied out-of-plane magnetic field, giving rise to a pseudoquantum Hall effect.^3,4^ Different from the real magnetic field, however, time-reversal symmetry is preserved with a pseudomagnetic field.^2^ As a result, the vector potential generated by the strain has opposite signs at the two K valleys, thus leading also to a valley Hall effect.^5,6^ Strain in graphene has been induced by either geometrical confinements such as in nanobubbles^7^ and ripples,^6,8^ nanoscale strain engineering,^9−12^ or lattice mismatch in heterostructures such as graphene/(NbSe_2_, BN)^13^ and graphene/black-phosphorus.^14^ A pseudomagnetic field up to 800 T has been reported.^12^ Beyond graphene, similar phenomena have also been predicted for other Dirac materials, including Dirac and Weyl semimetals,^15−19^ Weyl superconductors.^16,20−23^ However, experimental observations are limited to a recent report of strain-induced Landau levels on the surface of cleaved Weyl semimetal Rhenium-doped MoTe_2_,^24^ where a moderate 3 T field is reported. Furthermore, the strain-induced pseudomagnetic field is similarly calculated for kagome lattice, characterized by a two-dimensional hexagonal network of corner-sharing triangles, leading to linearly dispersing Dirac states at the K point and flat band through the rest of the Brillouin zone.^25^ There has been no experimental report of pseudomagnetic field in kagome materials.

Here, we provide strong evidence for strain-induced pseudomagnetic field over 1000 T in epitaxial FeSn films grown on the SrTiO_3_(111) (STO) substrate by molecular beam epitaxy (MBE). The model kagome magnet FeSn consists of alternatingly stacked 2D kagome Fe_3_Sn (K) and honeycomb Sn_2_ (S) layers (Figure 1a).^26−28^ The S-layer, or stanene, has a quasi-2D electronic structure,^26,29^ similar to graphene. However, the stanene’s Sn–Sn bond is weaker than the C–C bonding in graphene due to a larger bond length, thus facilitating a greater degree of its distortion or deformation. Using scanning tunneling microscopy/spectroscopy (STM/S), we show that the Sn honeycomb can be significantly distorted by epitaxial strain, leading to periodic stripe modulations with a periodicity of *l* = 2.0 nm, ∼3.8 *a*_FeSn_. Such modulations also result in differential conductance peaks consistent with pseudo-Landau levels generated by pseudomagnetic fields of over 1000 T. Our results demonstrate the feasibility of strain engineering topological magnets at the nanoscale.

**Figure 1:**
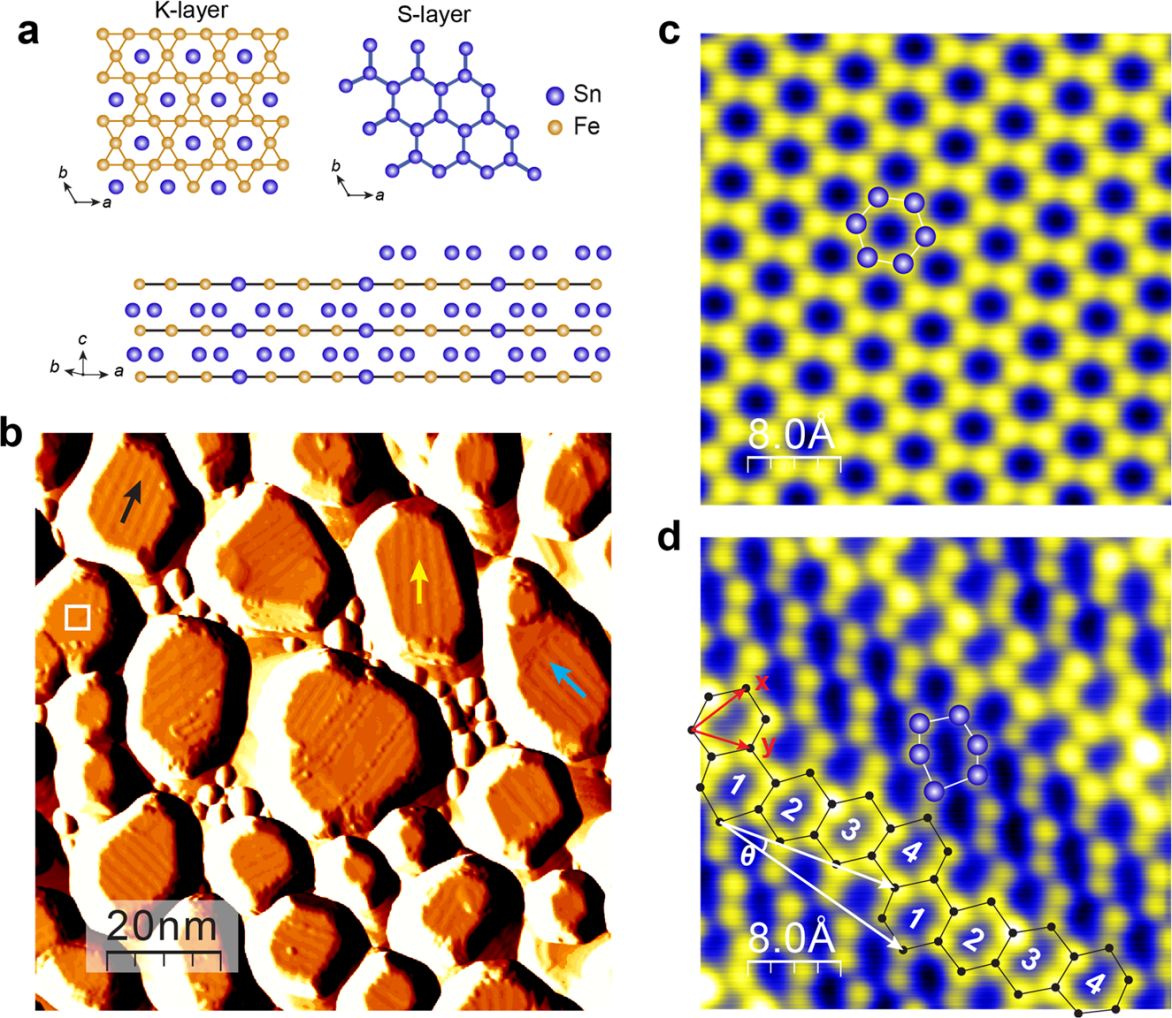
Molecular beam epitaxy growth of FeSn films on SrTiO_3_(111). a, Ball-and-stick model of the FeSn crystal structure from top and side views. b, Morphology of a FeSn film grown at *T*_sub_ = 480 °C, set point: *V* = 3.0 V, *I* = 10 pA. The STM image is in differential mode, and the height of the islands varies from to 6 to 7 nm. c, Atomic resolution STM image showing a perfect honeycomb lattice on the surface of a flat FeSn island, set point: *V* = −2.0 mV, *I* = 5.0 nA. d, Atomic resolution image revealing strongly distorted Sn honeycombs on the island with stripe modulations, set point: *V* = 20 mV, *I* = 5.0 nA.

## MBE Growth of FeSn Films on SrTiO_3_(111) Substrates

The FeSn films are grown on STO substrates, which are thermally treated *in situ* to obtain a flat-surface morphology with a (4 × 4) reconstruction^30^ (STM images of the annealed SrTiO_3_(111) are provided in Figure S1, Supporting Information). Due to an in-plane lattice mismatch between FeSn (*a*_FeSn_ = 5.30 Å)^31^ and SrTiO_3_(111) (*a*_STO(111)_ = 5.52 Å),^30^ a tensile strain ε = 3.99% is expected in epitaxial FeSn films. The growth follows the Volmer–Weber mode, i.e., island growth, at *T*_sub_ between 480 and 530 °C, characterized by three-dimensional flat-top islands as revealed by the topographic STM image in Figure 1b and line-profiles shown in Supporting Information Figure S2. The growth of the FeSn phase is confirmed by X-ray diffraction (XRD), which shows diffraction peaks of FeSn (002) and (021) planes (Supporting Information, Figure S3). As FeSn consists of vertically stacked Fe_3_Sn kagome and Sn_2_ honeycomb layers, there are two possible interfaces with the SrTiO_3_ substrate. An earlier study indicates a complex interface with Fe_3_Sn kagome layer on a Ti-rich termination layer of the SrTiO_3_ substrate.^32^ For FeSn films studied here, both surface terminations are observed (STM images of mixed termination and K-layer are shown in Supporting Information, Figures S4–5), with the Sn-termination the most common, likely due to Sn-rich growth conditions (Supporting Information Note 1). In the region outlined by the white square, atomic resolution imaging reveals a perfect honeycomb structure (Figure 1c), which is also independent of the bias voltage (Supporting Information, Figure S6).

The majority of the islands, however, exhibits periodic stripe modulations with an average periodicity of *l* = 2.0 nm, ∼3.77 *a*_FeSn_. A few examples are marked by black, cyan, and yellow arrows in Figure 1b. The stripes are distributed along three directions, ∼150° apart (Figure S7, Supporting Information). As shown in Figure S2, the stripes are commonly observed on islands less than 10 nm thick, which are likely more strained. For films grown at higher temperature of 530 °C, the island density is slightly reduced with larger lateral size and increased height, without stripe modulations on the surface. The fact that the thicker films are still under strain is further confirmed by *ex situ* XRD measurement, where the FeSn (002) and (021) peaks are slightly shifted to higher values, indicating smaller lattice constant in the *c*-direction, which is typically accompanied by an expansion of the in-plane constant. However, strain in these thicker films is likely not enough to distort the Sn or Fe_3_Sn layer.

## Strain-Induced Strong Distortion of the Sn Honeycomb Lattice

Atomic resolution imaging further reveals that the stripes arise from distortion of the Sn honeycomb lattice. As marked by the ball-and-stick model in Figure 1d, the building block is a group of four slightly distorted honeycombs periodically shifted by one-and-half unit bond length along the ***x***-direction, forming a stripe at θ = 14.3° with respect to the ***y***-direction. Between the stripes along the ***x***-direction, there are three strongly distorted honeycombs, where the middle unit exhibits the largest distortion (highlighted in white). The angles between the bonds (as marked) deviate significantly from the 120° of a perfect honeycomb.

**Figure 2:**
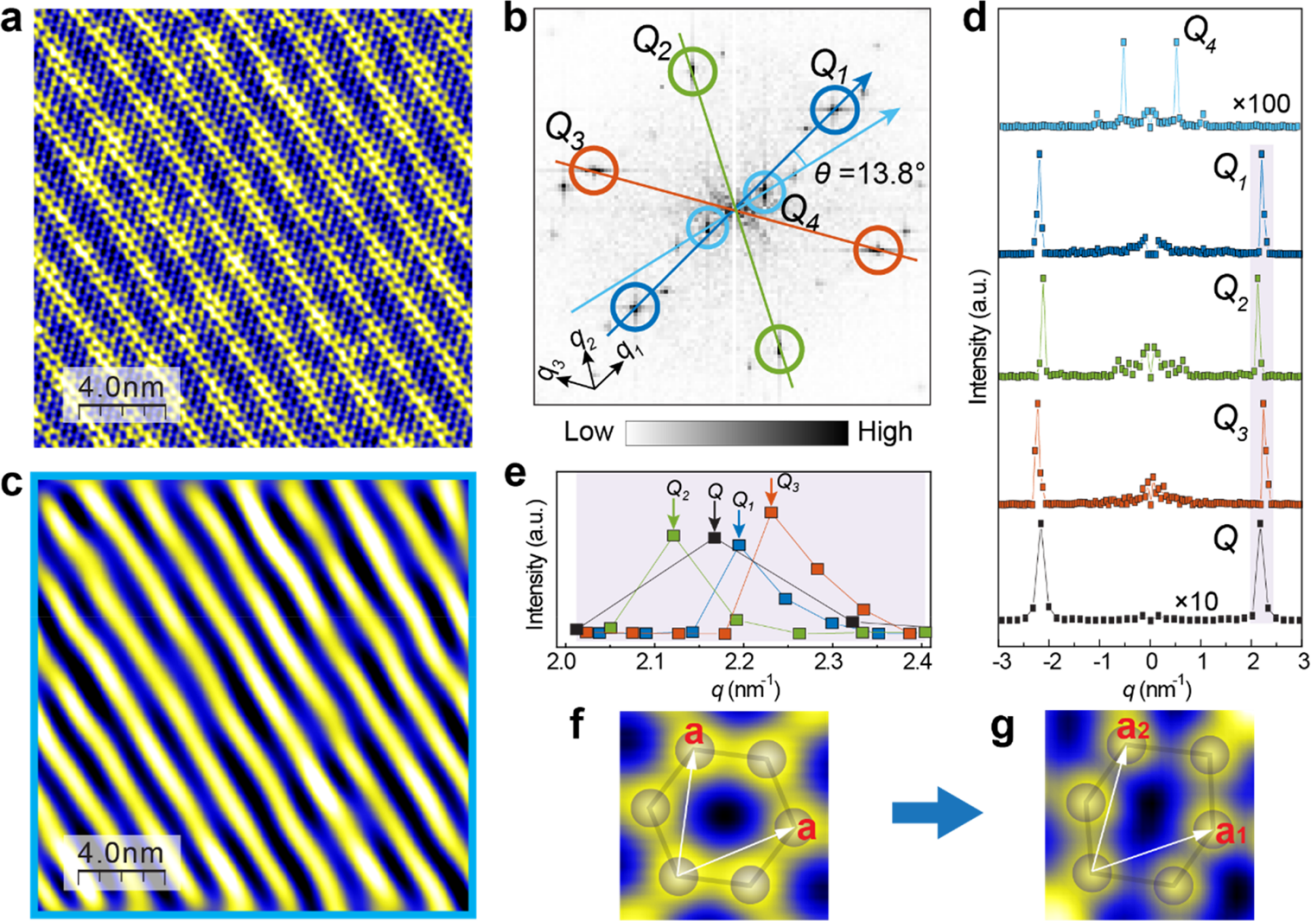
FFT analysis of the stripe modulations in strained FeSn/SrTiO_3_(111) films. a, STM topographic image of stripe modulations of Sn honeycomb lattice, set point: *V* = 100 mV, *I* = 5.0 nA. b, FFT of the image in (a). Bragg peaks along three directions ***q***_***1***_, ***q***_***2***_, and ***q***_***3***_ are denoted by *Q*_1_, *Q*_2_, and *Q*_3_ in blue, green and red circles, respectively. The diffraction peak of the stripe modulation is denoted by *Q*_4_ in cyan circles. There is an angle of 13.8° between Q_4_ and *Q*_1_ directions. c, Reverse-FFT of the peak *Q*_4_ in (b). The distribution of the stripes coincides with the topography in (a). d, Line profiles along the *Q*_1_, *Q*_2_, *Q*_3_ and *Q*_4_ directions in (b). As a reference, the line profile across the Bragg lattice *Q* is obtained from the FFT of a perfect honeycomb lattice. e, Close-up view of the Bragg peaks *Q*_1_, *Q*_2_, *Q*_3_, and *Q*. The peak position is marked by arrows in corresponding colors. f, Perfect honeycomb with lattice vector ***a*** marked. g, Deformed honeycomb with lattice vectors ***a***_***1***_ and ***a***_***2***_ marked.

The stripe formation is further confirmed by fast Fourier Transformation (FFT) analysis of STM images. An example is shown in Figure 2a, where the periodic stripe modulation of the Sn honeycomb is apparent. In addition to the Bragg peaks *Q*_1_, *Q*_2_, and *Q*_3_ characteristic of the honeycomb lattice, the fourth feature *Q*_4_ is also observed (Figure 2b), as the result of the stripe pattern, which is further confirmed by the selective reverse-FFT image (Figure 2c). The stripe forms a 13.8° angle with respect to *Q*_1_, consistent with that calculated from the real space image (Figure 1d). Furthermore, the Bragg peaks *Q*_1_, *Q*_2_, and *Q*_3_ exhibit different lengths compared to that of *Q* for a perfect honeycomb lattice (Figures 2d,e). For a quantitative comparison, calibration of the lattice distortion caused by the nonlinearity of the STM scanner is carried out first (Supporting Information Figures S8–S9). The results show that while *Q* corresponds to a lattice constant of 0.533 nm, the Bragg peaks *Q*_1_, *Q*_2_, and *Q*_3_ correspond to lattice constants of 0.526, 0.544, and 0.518 nm, respectively. This leads to an average 1.26% and 2.85% compressive strain along the ***q***_***1***_ and ***q***_***3***_ directions, respectively, and a 2.16% tensile strain along the ***q***_***2***_ direction. This analysis is consistent with that observed in real space. For example, the 2.16% tensile strain along ***q***_***2***_ direction is normal to the orientation of stripes (Figures 2a,b). Overall, the perfect honeycomb is likely deformed by the epitaxial strain, leading to different lattice vectors ***a***_***1***_ and ***a***_***2***_ (Figures 2f,g). These strain-induced distortions thus break the *C*_3_ symmetry of the stanene layer (albeit one direction is more pronounced), satisfying the conditions to generate pseudomagnetic fields.^3,33^

As STM imaging is a convolution of structural and electronic contributions, a natural question arises whether the distortion of the honeycomb lattice is structural or electronic. The stripe modulations indeed exhibit bias-dependence, with the distortion the most obvious at bias voltages closer to the Fermi level (Analysis of the bias-dependent stripe modulations is provided in Supporting Information Figures S10–12). While the relative amplitude varies from 8 to 20 pm depending on the imaging bias voltage, the feature associated with stripe modulations is always observed in the FFT patterns at all energies. We can also rule out the possibility of tip artifacts based on imaging of stripes across different domains (Supporting Information Figure S7). In addition, the stripe modulations are only observed on the Sn layer and not the Fe_3_Sn kagome layer. An example is shown in Supporting Information Figure S4 for an island with mixed S- and K-termination, where only the S-termination is distorted while a close-packed structure is intact on the K-termination. This is likely due to the weaker bonding in the S-layer facilitating greater lattice deformation. Overall, these observations indicate that the stripe modulations are primarily structural.

## Periodic Modulations of FeSn Electronic Structure

**Figure 3:**
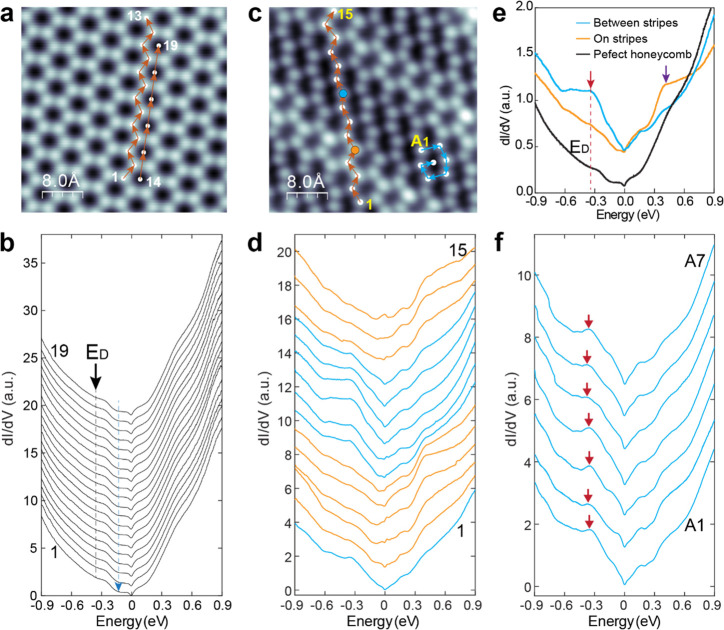
Modulations of the electronic structure in strained FeSn/SrTiO_**3**_(111) films. a, Atomic resolution image of perfect honeycomb lattice, set point: *V* = 3.0 V, *I* = 10 pA. b, A series of dI/dV spectra taken at sites marked in (a). The energy position of the Dirac point *E*_D_ is marked by the black arrow and dashed line, and the dip feature at −0.15 eV is marked by a cyan arrow. c, Atomic resolution image of distorted Sn honeycomb with stripe modulations, set point: *V* = 10 mV, *I* = 1.0 nA. d, A series of dI/dV spectra taken at sites marked in (c). The dI/dV spectra are classified into two groups, orange (taken on stripes) and cyan (taken between stripes). e, dI/dV spectra taken at sites on (orange) and between stripes (cyan), compared to the reference spectrum taken on perfect Sn honeycomb without stripe modulations. The pronounced peak at *E* = −0.36 eV (denoted with red arrow and dashed line) coincides with the Dirac point. f, dI/dV spectra taken within one-unit-cell of the strongly deformed honeycomb lattice, marked in (c). The energy position of the *E*_D_ is uniform within the deformed honeycomb lattice. The set point remains the same during the line dI/dV measurements.

For the perfect honeycomb lattice (Figure 3a), spatially uniform dI/dV spectra are observed (Figure 3b), characterized by three features: a gap near Fermi level, one dip at −0.15 eV (cyan arrow and dashed line), and another at −0.36 eV (black arrow and dashed line). The latter feature, with an averaged energy position of −0.36 ± 0.01 eV, is attributed to the Dirac point *E*_D_, similar to the −0.4 eV reported in recent photoemission studies on the Sn-terminated surface of cleaved bulk FeSn.^26,27^ The slight shift of the Dirac point in FeSn/STO films relative to the bulk value is likely due to charge doping from the SrTiO_3_(111) substrate. While the origins of the gap near the Fermi level and dip at −0.15 eV are unknown, all three features are modulated by the formation of stripes as revealed by spatially resolved differential dI/dV spectroscopy discussed below.

Figure 3d shows a series of dI/dV spectra taken at sites 1–15 marked in Figure 3c. Spectra taken on the stripe (weakly distorted honeycomb) and between stripes (strongly distorted honeycomb) are further highlighted in Figure 3e. Compared to the perfect honeycomb lattice, a new peak at ∼0.4 eV above *E*_F_ (purple arrow in Figure 3e) appears for the weakly distorted honeycomb (on stripe). Below *E*_F_, a pronounced peak around *E*_D_ = −0.36 eV emerges for the strongly distorted honeycombs (between the stripes) (red arrow in Figure 3e). The same feature is consistently observed at all seven characteristic sites within the strongly distorted honeycomb (Figure 3f), which suggests that the electronic modulation follows the structural modulation, extending beyond the individual unit cell of the honeycomb, and consistent with the stripe distribution. For dI/dV spectra taken on stripes, slight spatial variation is observed (additional dI/dV spectra provided in Figure S13). The dI/dV spectra are also independent of the tunneling current *I*. The line shape of the dI/dV spectra remains similar as current is varied by almost 2 orders of magnitude from 0.1 to 7.0 nA, with only variations in spectral intensity (Supporting Information, Figure S14). Note that the gap Δ ∼ 7.9 meV at *E*_F_ is observed on the Sn-terminated surface without (Figure 3b) or with stripe modulations (Figures 3d,f). Therefore, it is unlikely a charge density wave gap. This is further confirmed by systematic analysis of energy-dependent dI/dV maps where the intensity of the stripe modulation is uncorrelated to this gap (Supporting Information, Figure S15). The origin of the gap is unknown at the moment, but may be due to electron–electron interactions, as that observed in 1T′-WTe_2_.^34^

## Periodic Strain-Induced Pseudomagnetic Field

**Figure 4:**
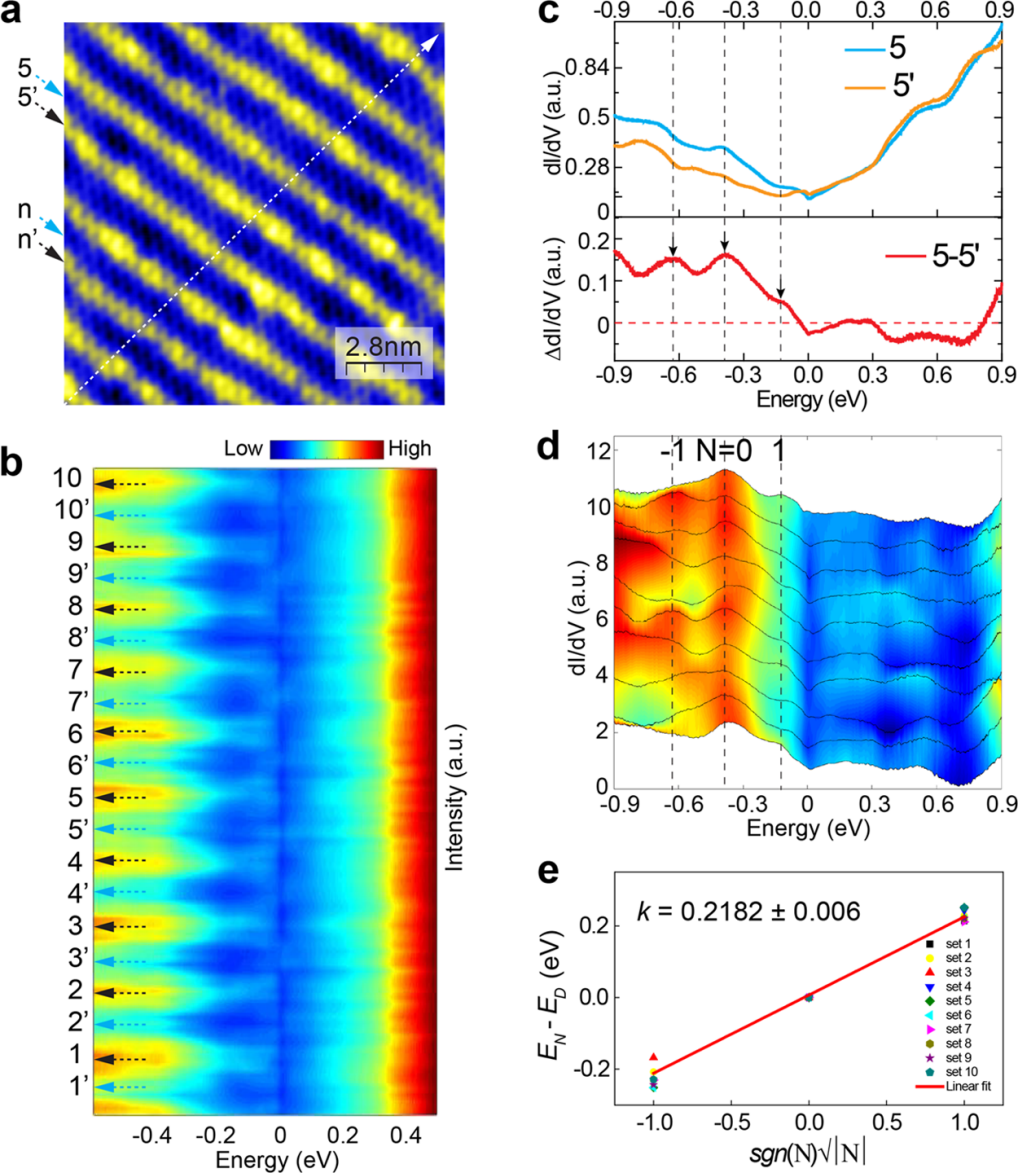
Periodic pseudomagnetic fields induced by stripe modulations in strained Sn-terminated FeSn/SrTiO_3_(111) films. a, STM topographic image showing the stripe modulations, where the on and between stripe sites are labeled as n′ and n, respectively. Set point: *V* = 1.0 V, *I* = 7.0 nA. b, dI/dV spectra taken along the white arrow in (a) with the intensity displayed in false color. The set point remains the same during the line dI/dV measurements. c, Upper panel: dI/dV spectra taken on stripe (labeled 5′ in (a)) and between stripes (5 in (a)). Lower panel: difference spectrum with three peaks marked by black arrows. d, Difference dI/dV spectra by subtracting n and n′. The most pronounced peak at −0.36 eV is assigned to *N* = 0th Landau level and the neighboring two peaks are assigned to *N* = ± 1st. e, Plot of peak energy (*E*_N_ – *E*_D_) vs *sgn*(*N*)√|*N*|. The linear fitting (red line) yields to a slope *k* = 0.2182 ± 0.006.

The periodic stripe modulations also lead to regularly modulated electronic properties. Figure 4a shows a region with ten periodic stripe modulations, where n′ and n label the on or between stripe sites. Figure 4b is a false color plot of a series of dI/dV spectra taken along the white arrow in Figure 4a (dI/dV spectra are shown in Figure S16, Supporting Information). Periodic variations of the differential conductance are clearly resolved, consistent with the topographic stripe modulations in Figure 4a. To quantitatively determine the modification in electronic structures induced by strain, differential spectra on and between stripe sites (n-n′) are obtained. An example is shown in Figure 4c, which reveals three peaks marked by black arrows and dashed lines (lower panel). A similar analysis was done for the rest of the spectra (Figure 4d), where the same peaks are also observed as marked by black dashed lines (Figure S17, Supporting Information).

The appearance of these conductance peaks could be due to quantum well states from spatial confinement,^35^ given that the islands are mostly less than 100 nm in diameter (c.f. Figure 1b). However, periodic modulations of the dI/dV spectra were only observed on islands that are less than 10 nm in height, but not on taller islands of comparable size (Supporting Information Figure S2). On the latter type of islands, bias-dependent imaging also did not reveal any modulations (c.f. Supporting Information Figure S6). Another possible mechanism is defect-induced states. The most commonly observed defects are Sn divacancy and substitutional defects, as shown in Supporting Information Figure S18, both of which do induce bound states appearing as peaks in dI/dV spectra. However, their line shapes, particularly the single peak position at −86 and −61 meV, respectively, are intrinsically different from those caused by the stripe modulations.

After ruling out these possible mechanisms, we attribute the conductance peaks to quantized Landau levels (LLs) originating from the strain-induced pseudomagnetic field. Such field is a general response of materials with linear energy dispersion to strain, which generates axial vector and scalar potentials.^4^ At the level of tight binding, the vector potential will modify the massless Dirac Hamiltonian as follows:^4^
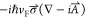
1similar to that from a real magnetic field. This will lead to LLs that are expected to follow:^36,37^
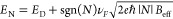
2Here, *E*_D_ is the energy of Dirac point, ν_F_ the Fermi velocity, *e* the electron charge, ℏ the reduced Planck constant, and *B*_eff_ the pseudomagnetic field. The integer *N* represents an electron-like (*N* > 0) or a hole-like (*N* < 0) LL index.

For the dI/dV spectra shown in Figure 4d, the most pronounced peak at *E* = −0.36 eV coincides with the Dirac point *E*_D_, and is assigned to the zeroth Landau level (labeled *N* = 0). Starting from this charge neutrality point *N* = 0 LL, the neighboring two LLs are labeled as *N* = ± 1. The plot of *E*_N_ – *E*_D_ as a function of  is shown in Figure 4e, which follows the unique square root dependent sequence, confirming the expected scaling behavior for LLs. The linear fit yields a slope *k* = 0.2182 ± 0.006. To calculate the value of the pseudomagnetic field, Fermi velocity is needed. Since Fermi velocity for FeSn thin films is not available, we adopt the values for bulk FeSn of ν_F_ = (1.7 ± 0.2) × 10^5^ ms^–1^,^26^ which yields an effective pseudomagnetic field of *B*_eff_ = 1251 ± 363 T (details in Supporting Information Note 2). Note that a recent work^32^ shows that ν_F_ can depend strongly on the interlayer coupling between the kagome and the stanene layers in FeSn thin films, where a significant decrease of ν_F_ can be expected at the bilayer limit. This suggests a low limit for our estimated effective pseudomagnetic field. On the other hand, a lower limit can be obtained by using the Fermi velocity of free-standing stanene^38^ (see Supporting Information Note 2 for details).

While pseudomagnetic fields up to 800 T had been previously reported in graphene in nanobubbles or nanocrystals,^7,13^ our results show a periodic pseudomagnetic field in excess 1000 T in strained kagome magnet FeSn/SrTiO_3_(111) films grown at temperatures below 500 °C. Moreover, unlike the localized nature of the geometrical deformation in most graphene nanostructures, the pseudomagnetic fields found here distribute periodically across the surface. This can be clearly seen in the differential conductance map *g*(**r**, −300 meV) near the zeroth LL with periodic higher (lower) contrast for the regions between the stripes (on the stripes) (c.f. Supporting Information Note 3 and Figure S19). Similar behavior is found for multiple samples as shown in Supporting Information Figures S20–21, where the induced pseudomagnetic field is also independent of the stripe orientations.

The large pseudomagnetic field can be attributed to several factors. First, the Sn–Sn bond length in single layer Sn honeycomb lattice, or stanene, is predicted to be 2.87 Å, the longest among all group IV honeycomb lattices, and two times larger than that of the C–C bond length in graphene (1.42 Å).^39^ The longer Sn–Sn bond length means a weaker π–π bond, which can facilitate the formation of a buckled structure with a mixed character of sp^2^ and sp^3^ orbital hybridization.^39^ This makes the synthesis of freestanding stanene challenging, and most films synthesized to date exhibit a close-packed structure due to the vertical displacement of Sn atoms.^40^ On cleaved surfaces of kagome materials, the formation of a perfect Sn honeycomb lattice depends on the stacking order of the Sn and kagome layers. For CoSn consisting of alternating Sn and Co_3_Sn layers,^41^ a perfect honeycomb structure was seen, while a buckled honeycomb structure was observed on Fe_3_Sn_2_ where the Sn layer is separated by two Fe_3_Sn kagome layers.^42,43^ Here, our comprehensive STM/S studies of multiple FeSn films epitaxially grown on SrTiO_3_(111) substrates have revealed a perfect Sn honeycomb lattice for those grown at temperature *T* = 530 °C. At lower temperatures, the stripes are commonly observed on the Sn-terminated FeSn islands with thickness below 10 nm, attributed to epitaxial strain resulting in a distorted honeycomb lattice. These findings indicate that in addition to uniform buckling, the Sn honeycomb can also form long-range deformation for strain-relief, highlighting the significant tunability of epitaxial kagome thin films. Buckling of the honeycomb structure has also been found to be critical in the 2D magnetism of silicene and germaneness in Zintl phase compounds,^44,45^ indicating that strain engineering can be a fertile ground for tuning these types of materials for spintronic applications.

In summary, we have grown thin films of kagome magnet FeSn on the SrTiO_3_(111) substrates by MBE and observed strain-induced periodic modulations of the Sn honeycomb lattice for film thickness less than 10 nm. Such modulations lead to periodic differential conductance peaks consistent with Landau levels generated by pseudomagnetic fields greater than 1000 T. Our findings demonstrate a viable path toward strain engineering electronic properties of kagome materials.

## Methods

### Sample Preparation

The FeSn films were grown by MBE on Nb-doped (0.05 wt %) SrTiO_3_(111) substrates, which were were first degassed at 600 °C for 3 h, and then followed by annealing at 950 °C for 1 h to obtain a flat surface with step-terrace morphology. During the MBE growth, high purity Fe (99.995%) and Sn (99.9999%) were evaporated from Knudson cells setting at 1150 and 805 °C on the SrTiO_3_(111) substrate at temperatures between 480 to 530 °C. The Fe/Sn ratio is estimated to be 1:2.7 during growth.

### LT-STM/S Characterization

The STM/S measurements were carried out at 4.5 K in a Unisoku ultrahigh vacuum low-temperature STM system. Polycrystalline PtIr tips were used and tested on Ag/Si(111) films before the STM/S measurements. The dI/dV tunneling spectra were acquired using a standard lock-in technique with a small bias modulation *V*_mod_ = 20 mV at 732 Hz.

## Supporting Information Available

MBE growth of FeSn films on SrTiO_3_(111) substrate; Calculations of the pseudomagnetic field; Modulation of electronic properties by stripes revealed by dI/dV maps; STM imaging of the annealed SrTiO_3_(111) substrate; Initial stages of FeSn growth on SrTiO_3_(111) substrates; X-ray diffraction (XRD) data of a 20 nm FeSn/SrTiO_3_(111) film; STM imaging of the mixed Sn termination (S layer) and Fe_3_Sn kagome termination (K layer) in FeSn/STO(111) films; Bias-dependent STM imaging of the kagome layer on the K-terminated FeSn/SrTiO_3_(111) film; Bias-dependent STM imaging of the Sn honeycomb on the Sn terminated FeSn/SrTiO_3_(111) film; Stripe modulations on the Sn-terminated FeSn/SrTiO_3_(111) films; FFT of perfect and deformed honeycomb lattices; Calibration of lattice distortion caused by nonlinearity of the STM scanner; Bias-dependent STM imaging of distorted honeycomb lattice; Bias-dependence of stripe modulations; FFT of bias-dependent STM images; Site-dependent dI/dV spectra within one honeycomb lattice; Set point-dependent dI/dV spectra on stripe (n′) and between stripes (n); FFT analysis of the stripe modulations; Periodic stripe modulations; Subtraction of 10 sets of dI/dV spectra to reveal the Landau levels; Bound states associated with two types of defects on the Sn-terminated FeSn/STO(111) film; Spatial-resolved dI/dV maps; Another example of Landau levels by pseudomagnetic field; Laudau levels independent of the stripe orientations (PDF)
