# Supplementary material for: Giant Periodic Pseudomagnetic Fields in Strained Kagome Magnet FeSn Epitaxial Films on SrTiO3(111) Substrate
Source: Nano Lett. 2023 Mar 13;23(6):2397–404. doi: 10.1021/acs.nanolett.3c00345 (PMC10037333; doi:10.1021/acs.nanolett.3c00345)
Supplement: Supplementary file 1 — nl3c00345_si_001.pdf [file nl3c00345_si_001.pdf]

Supporting Information

Giant periodic pseudo-magnetic fields in strained kagome magnet FeSn epitaxial films on $SrTiO_3$(111) substrate

Huimin Zhang[1,2], Michael Weinert[3],and Lian Li[1*]

[1]Department of Physics and Astronomy, West Virginia University, Morgantown, WV 26506, USA

[2]State Key Laboratory of Structural Analysis, Optimization and CAE Software for Industrial Equipment, Dalian University of Technology, Dalian, 116024, China

[3]Department of Physics, University of Wisconsin, Milwaukee, WI 53201, USA

[*]Correspondence to: lian.li@mail.wvu.edu

This file includes:

Note 1: MBE growth of FeSn films on $SrTiO_3$(111) substrate

Note 2: Calculations of the pseudo-magnetic field

Note 3: Modulation of electronic properties by stripes revealed by dI/dV maps

Figures S1-S21

Note 1: MBE growth of FeSn films on $SrTiO_3$(111) substrate

The FeSn films were grown by molecular beam epitaxy (MBE) on Nb-doped (0.05 wt.%) $SrTiO_3$(111) substrates. The $SrTiO_3$(111) substrates were first degassed at 600 °C for 3 hours and then followed by annealing at 950 °C for 1 hour to obtain a flat surface with step-terrace morphology. During the MBE growth, high purity Fe (99.995%) and Sn (99.9999%) were evaporated from Knudson cells setting at 1150 °C and 805 °C and co-deposited on the $SrTiO_3$(111) substrate at a substrate temperature (T_{sub}) between 480 to 530 °C. The Fe/Sn ratio is estimated to be 1:2.7 during growth. The substrate temperature is monitored by a LAND portable infrared thermometer (Cyclops 160L). At T_{sub} = 480 °C, the surface of the film exhibits stripe modulations while at elevated T_{sub} = 530°C, such modulations are mostly absent, as shown in Figure S3. Multiple samples (> 5) were grown with these parameters.

FeSn consists of alternating Sn_2 honeycomb (Sn termination) and Fe_3Sn kagome layer (Fe_3Sn termination) vertically stacked. The stripe modulation only appears on Sn termination, while the Fe_3Sn termination exhibits the close-packed structure without noticeable distortion (Figure S5). The striped surface shows crest and trough morphology with a height contrast ~20.9 pm (c.f., Figure S10 for bias-dependent behavior). At atomic scale, the valley region (on the stripe) is of slight distortion while the trough region exhibits strong distortion, as shown with the ball-and-stick model imposed on top (Figure 1**g**).

Note 2: Calculations of the pseudo-magnetic field

In general, the response of Dirac materials, i.e., materials whose low energy excitations are described by massless Dirac equation, to strain is well understood[1]. For a honeycomb lattice, strain induced distortion will lead to axial vector and scalar potentials, which will strongly modify its electronic properties[2]. At the level of tight binding model, the potential will modify the Dirac Hamiltonian:

$$-i\hbar v_F \vec{\sigma}(\nabla - i\vec{A})$$

where $A_x = \frac{\sqrt{3}}{2}(t_3 - t_2)$ and $A_y = \frac{1}{2}(t_2 + t_3 - 2t_1)$, and t_i are the nearest neighbor hoping parameters. For a perfect honeycomb lattice, the t_i are the same, resulting in zero A. For distorted honeycomb, however, the lattice distortion will modify the lattice constant, thus the hopping

parameter, leading to non-zero A. Furthermore, assuming that the atomic displacements u_i are small in comparison with the lattice constant, then the hopping parameter can be written as:

$t_i = t + \frac{\beta t}{a^2}\vec{\delta}_i(\vec{u}_i - \vec{u}_0)$ and $(\vec{u}_i - \vec{u}_0) \propto (\vec{\delta}_i \nabla)\vec{u}(\vec{r})$ ($\vec{\delta}_i$ is the nearest neighbor vectors).

This leads to an effective magnetic field $B_{eff} = \nabla \times \vec{A}$, with

$$A_x = c\frac{\beta t}{a}(u_{xx} - u_{yy}),\ A_y = -c\frac{2\beta t}{a}u_{xy}$$

This effective field will result in Landau levels (LLs) that are expected to follow the equation, same as the one caused by the application of real electromagnetic fields[3,4]:

$$E_N = E_D + sgn(N)v_F\sqrt{2e\hbar|N|B_{eff}} \qquad (1)$$

Here, E_D is the energy of Dirac point, ν_F is the Fermi velocity, e is the electron charge $1.602 \times 10^{-19}\ C$, $\hbar$ is the reduced Planck constant $1.0546 \times 10^{-34}\ J \cdot$ s, and B_{eff} is the pseudo-magnetic field. The integer N represents an electron-like ($N > 0$) or a hole-like ($N < 0$) LL index.

$$k = \frac{E_N - E_D}{sgn(N)\cdot\sqrt{N}} \qquad (2)$$

The slope k is obtained from experimental data, as shown in Fig. 4**e** where $E_N - E_D$ is plotted as a function of $sgn(N)\sqrt{|N|}$. The linear fit in Fig. 4**e** yields to a slope $k = 0.2182 \pm 0.006$ eV.

$$B_{eff} = \frac{k^2}{2e\hbar v_F{}^2} \qquad (3)$$

Basing on Equation 3, we can calculate the effective pseudo-magnetic field B_{eff}. If we take the bulk FeSn value $\nu_F = (1.7 \pm 0.2)\times 10^5\ ms^{-1}$ due to lack of data for epitaxial FeSn films, the pseudo-magnetic field is determined to be B_{eff} = 1251 ± 363 T. If we take the Fermi velocity of stanene ($\nu_F = 4.4 \times 10^5\ ms^{-1}$)[5], the effective pseudo-magnetic field B_{eff} = 187 ± 10 T is obtained. In addition, the dI/dV spectra in Sn layer of FeSn (Fig. 3**b**) are intrinsically different from that of stanene on Au(111)[6] or Sb(111)[7]. Therefore, to adopt the Fermi velocity of FeSn is more appropriate.

The LLs for strain-induced pseudomagnetic field in kagome layer are expected to follow similar equation, however with an anisotropic Fermi velocity[8].

Note 3: Modulation of electronic properties by stripes revealed by dI/dV maps

The Sn honeycomb with stripe modulations are investigated by dI/dV mapping. The topographic image and its corresponding spatial dI/dV maps are displayed in Fig. S17**a**. Accompanied the stripes in the topography, we observe stripy electronic modulation across the surface in the dI/dV maps within the energy range [-500 meV, 500 meV]. Figure S17**b** compares the line profile derived from the topography and corresponding dI/dV maps, where the dashed red lines mark the valley position from the topography. Between the energy ranges [-500 meV, -100 meV] and [100 meV, 300 meV], the valley position in the topography corresponds to higher density of states. At Fermi level $g(\mathbf{r}, 0 \text{ meV})$, the modulation is almost gone. At the energy window [400 meV, 500 meV], the valley position in the topography corresponds to lower density of states. In other words, there is a π-phase shift between 400 meV and 500 meV, compared to the other energies.

We notice that there are different stripe orientations for different FeSn islands (Figure S6). dI/dV spectra and dI/dV maps are carried out for stripes with different orientations and we find that the orientation of the stripes has no detectable effect on the dI/dV spectra or maps. As shown in Figure S19, we find that not only the dI/dV spectra but also the spatial modulation is consistent along different orientations.

Supporting Figures

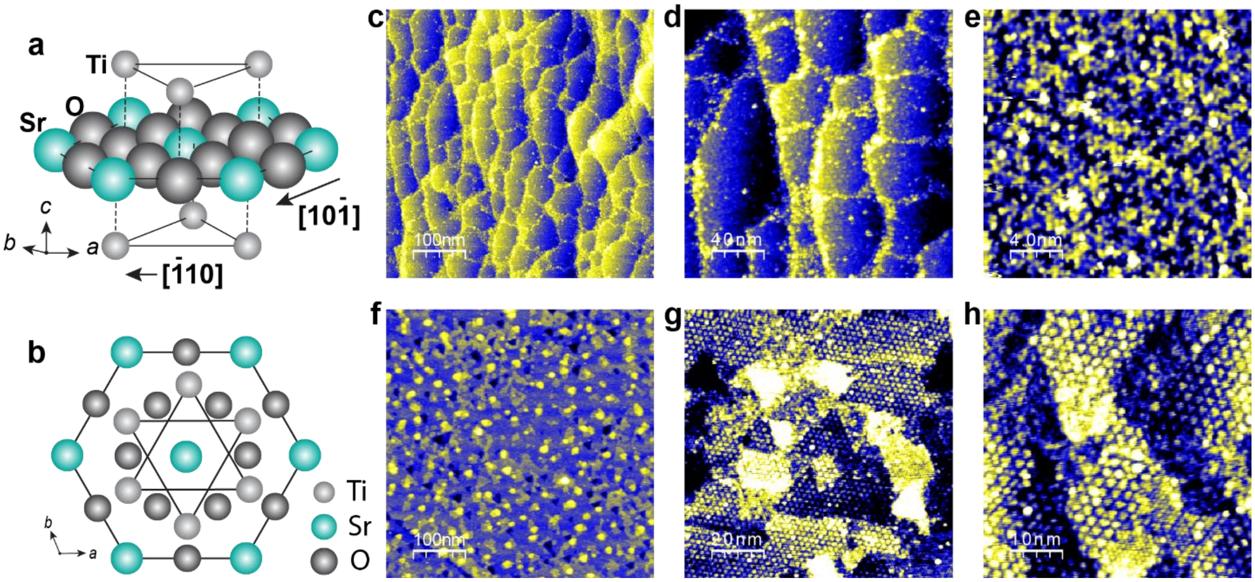

Figure S1. STM imaging of the annealed $SrTiO_3$(111) substrate. Ball-and-stick model of the $SrTiO_3$(111) surface: side view (**a**) and top-view (**b**). **c**-**e**, Topographic STM images of type-1 $SrTiO_3$(111) substrate. Setpoint: V = 3.0 V, I = 30 pA (c)-(d), and V = 1.0 V, I = 500 pA (**e**). **f**-**h**, Topographic STM images of type-2 $SrTiO_3$(111) substrate. Setpoint: V = 2.0 V, I = 10 pA for (**f**)-(**g**), and V = 2.0 V, I = 30 pA for (**h**). The $SrTiO_3$(111) surface exhibits a (4 $\times$ 4) reconstruction. The growth of FeSn films is similar on both types of $SrTiO_3$(111) substrates.

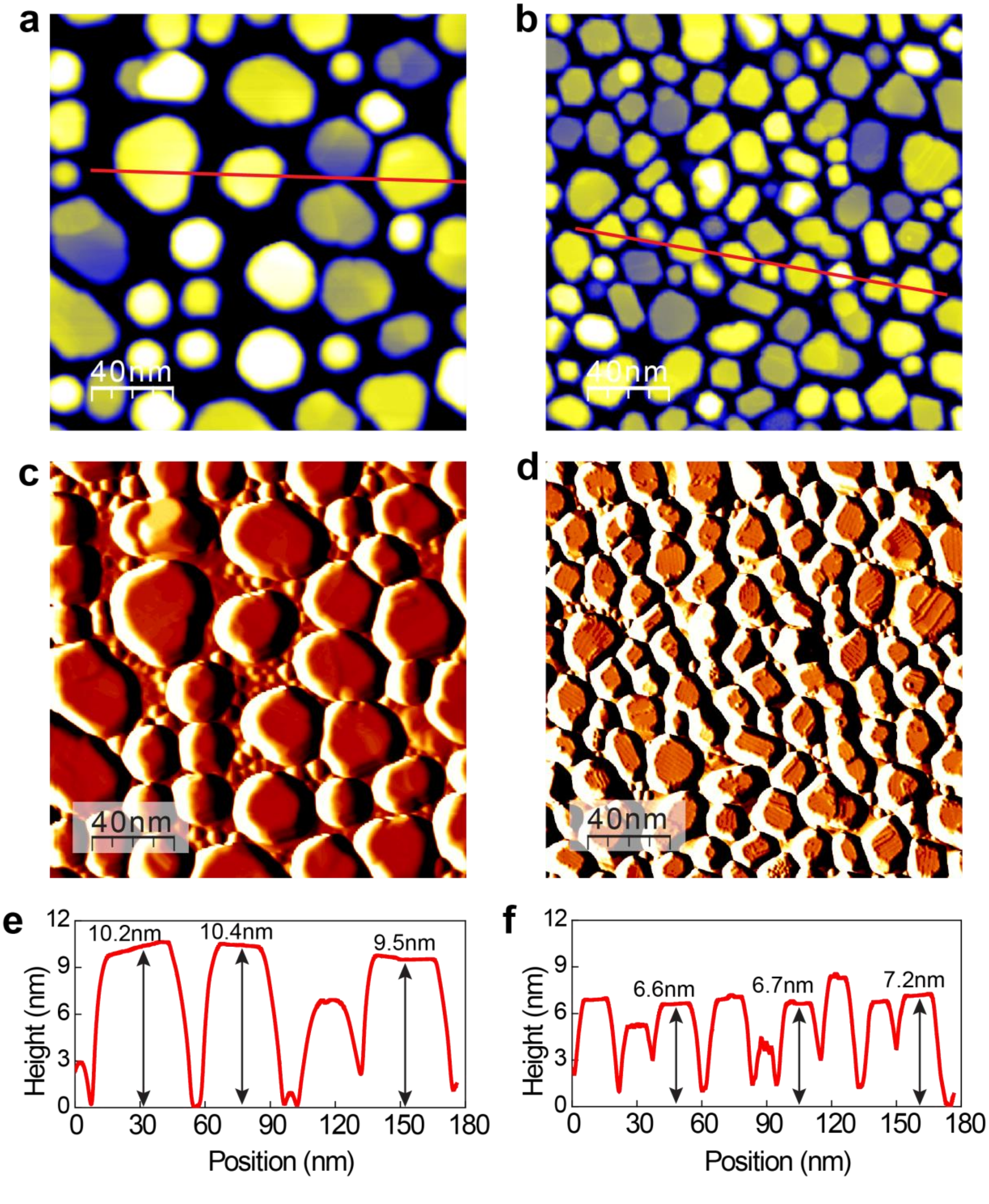

Figure S2. Initial stages of FeSn growth on $SrTiO_3$(111) substrates. **a**-**b**, STM topographic images of FeSn/$SrTiO_3$(111) films. Setpoint: V = 2,0 V, I = 10 pA. **c**-**d**, STM images of (**a**) and (**b**) in differential mode. **e**-**f**, Line profiles along the red line in (**a**) and (**b**), respectively.

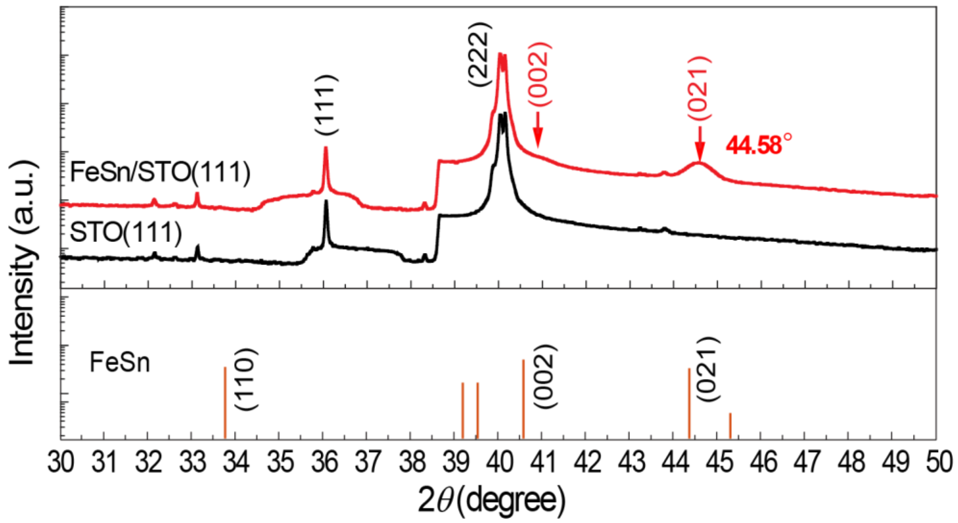

Figure S3. X-ray diffraction (XRD) data of a 20 nm FeSn/$SrTiO_3$(111) film. The peaks at 2θ = 40.52° and 44.58° are indexed as FeSn (002) and (021), confirming the FeSn phase. Note that both peaks are slightly shifted to higher values, indicating smaller lattice constant in the *c*-direction.

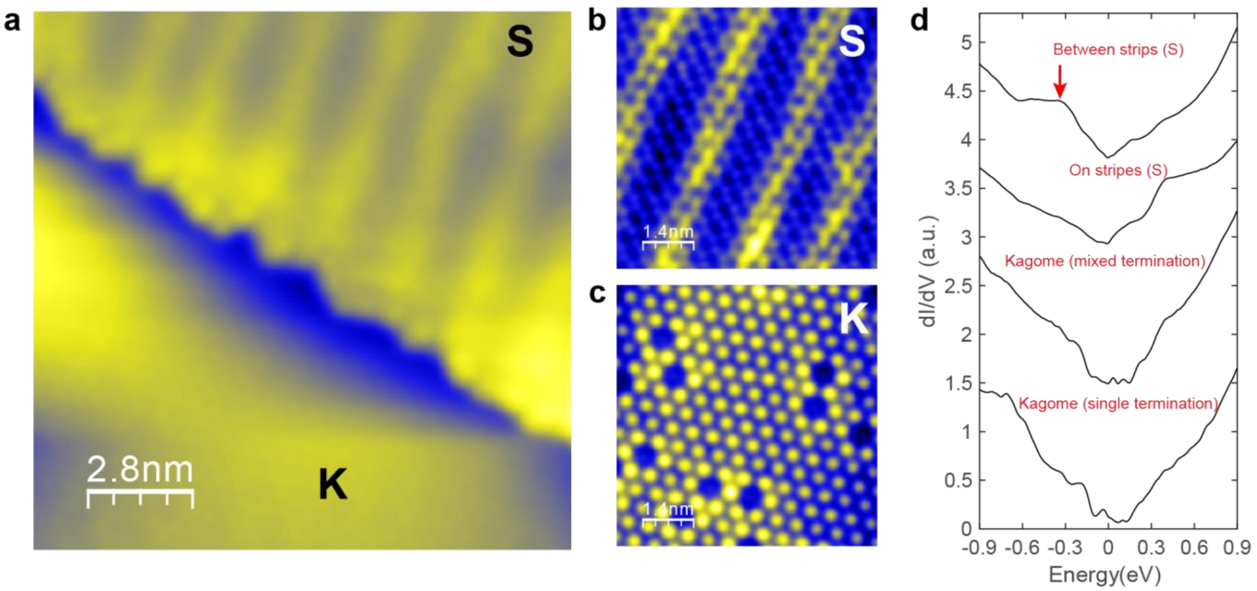

Figure S4. STM imaging of the mixed Sn termination (S layer) and Fe_3Sn kagome termination (K layer) in FeSn/STO(111) films. a, Topographic STM image showing both S and K terminations. Setpoint: V = 0.9 V, I = 3.0 nA. **b**, The STM image of the S-layer with stripe modulations. Setpoint: V = -0.1 V, I = 3.0 nA. **c**, STM image of the K-layer without stripe modulations. Setpoint: V = 0.2 V, I = 1.0 nA. **d**, Typical dI/dV spectra taken between stripes and on stripes on the Sn layer; and on single and mixed K layers.

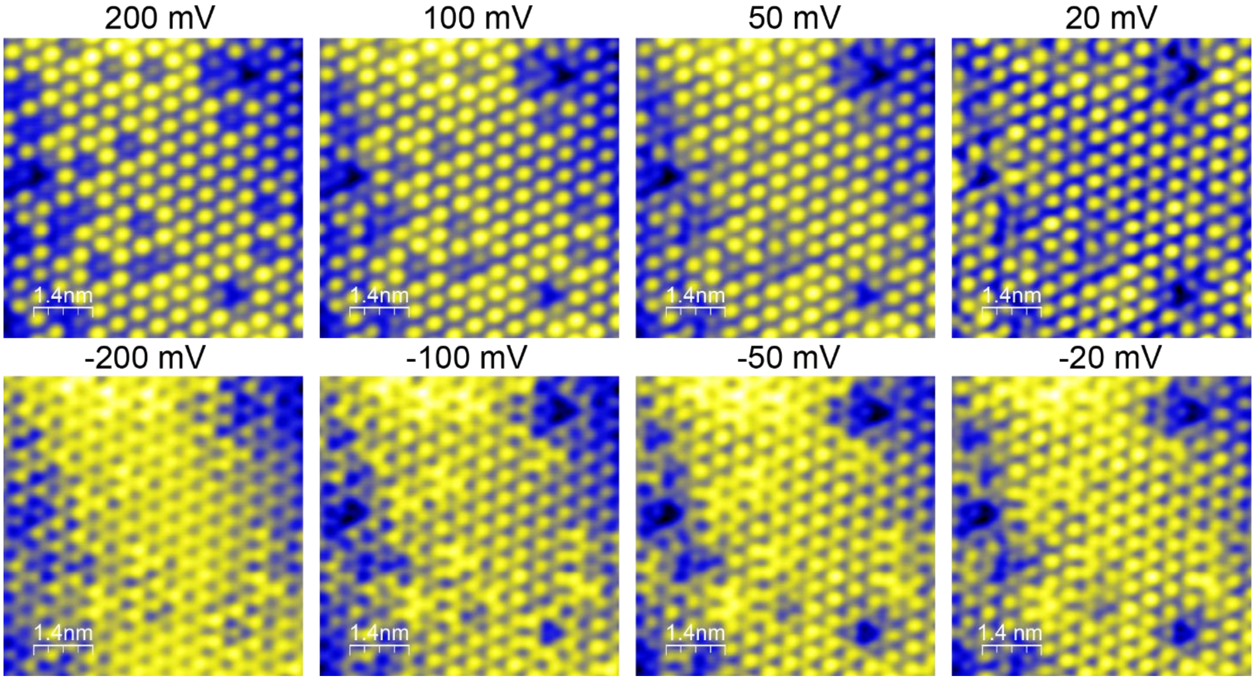

Figure S5. Bias-dependent STM imaging of the kagome layer on the K-terminated FeSn/SrTiO$_3$(111) film. Setpoint: I = 5.0 nA with V specified.

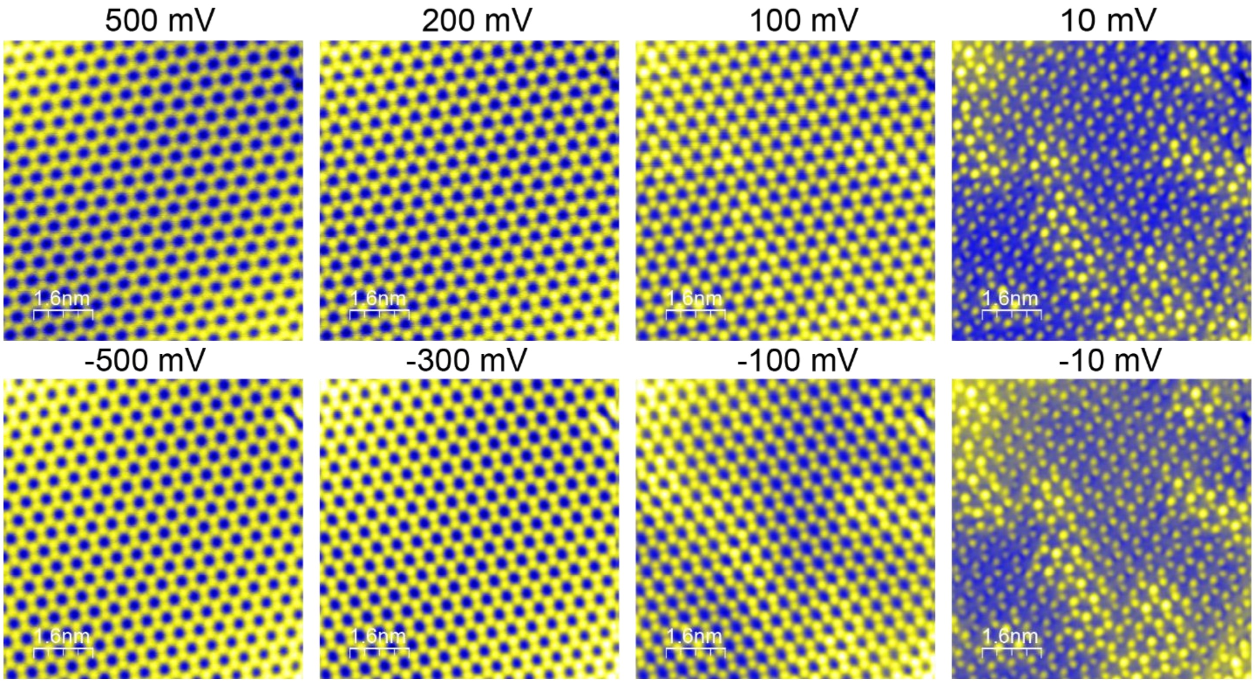

Figure S6. Bias-dependent STM imaging of the Sn honeycomb on the Sn terminated $FeSn/SrTiO_3(111)$ film. Setpoint: I = 5.0 nA with V specified. The defect states are revealed at -10 and 10 mV.

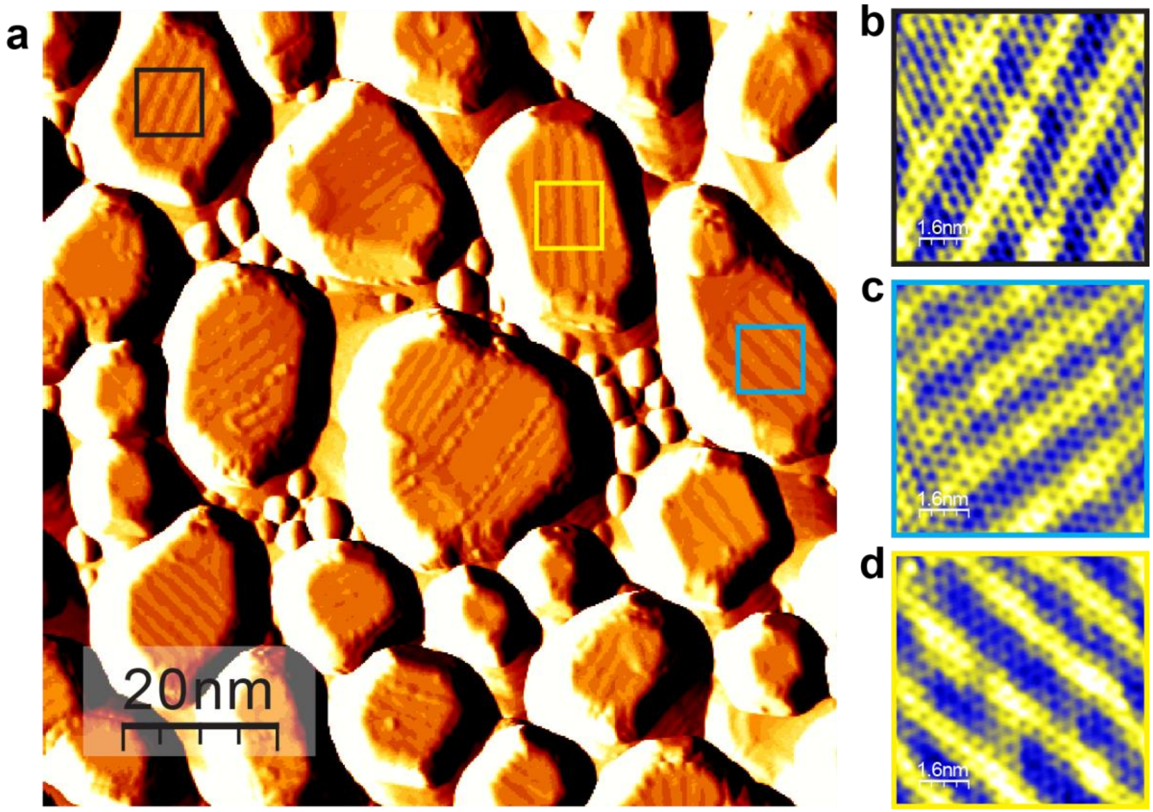

Figure S7. Stripe modulations on Sn terminated FeSn/SrTiO$_3$(111) films. a, STM image of a FeSn film grown on SrTiO$_3$(111) substrate in differential mode, setpoint: *V* = 1.0 V, *I* = 100 pA. **b**-**d**, STM images of the stripe modulations taken in the regions outlined by black, cyan, and yellow squares in (**a**). Setpoint: *V* = 3.0 mV, *I* = 5.0 nA.

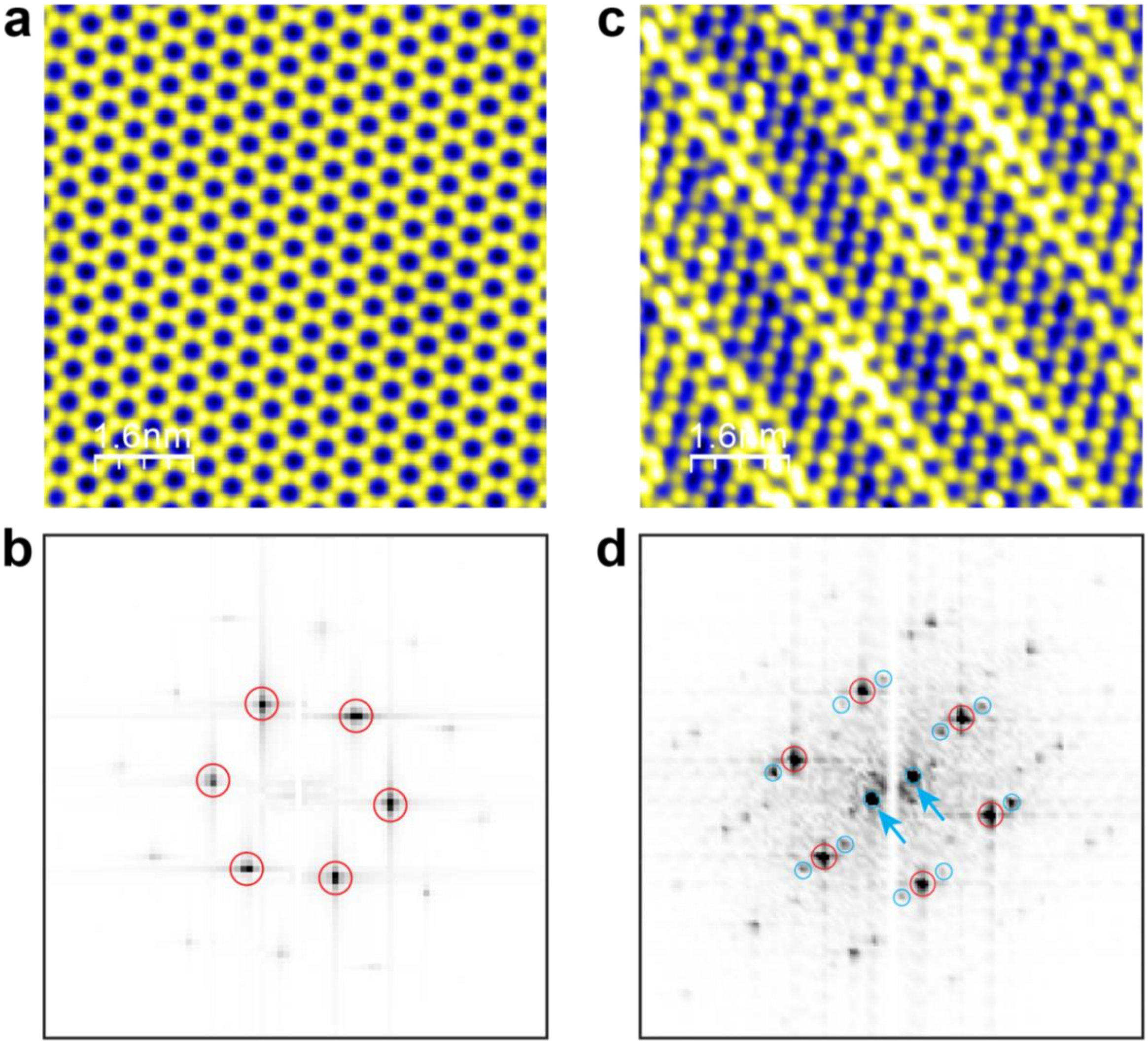

Figure S8. FFT of perfect and deformed honeycomb lattices. **a**, Atomically resolved STM image of the deformed honeycomb lattice, setpoint: V = 100 mV, I = 5.0 nA. **b**, FFT of (**a**) with Bragg peaks Q_1, Q_2 and Q_3 marked in red circles. The Q_4 in cyan circle denotes the stripe modulation. **c**, Atomic resolution image of a perfect honeycomb lattice, setpoint: V = -2.0 mV, I = 5.0 nA. **d**, FFT of (**c**). The red circles mark the Bragg peak Q.

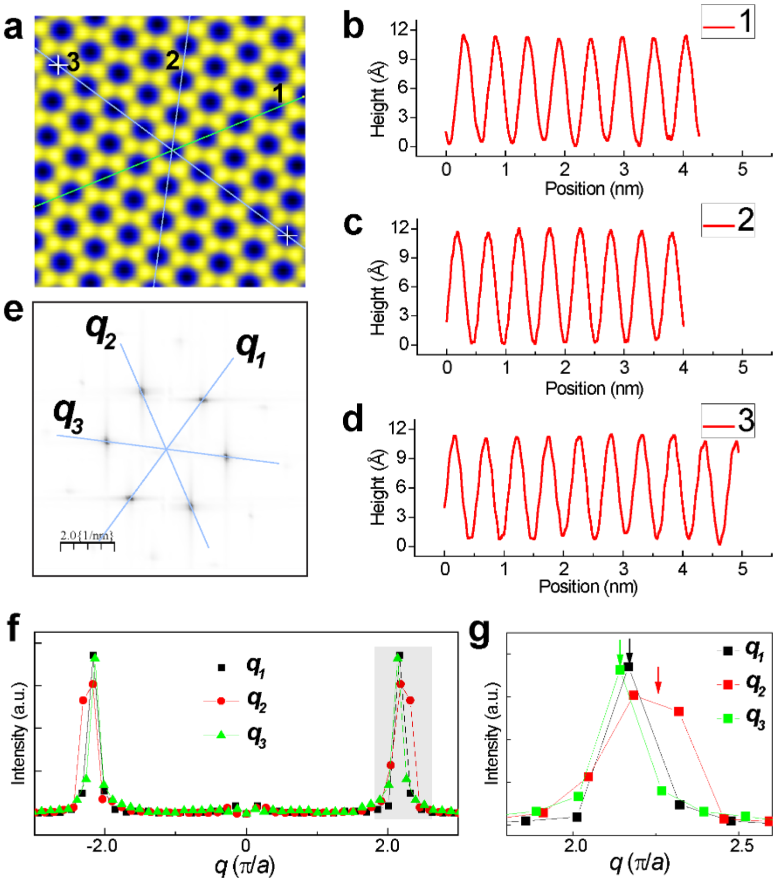

h

	Length (nm^{-1})	Lattice constant *a* (nm)	*q'*	Parameter α
q_1	2.1674745	0.5328	1	1
q_2	2.142064	0.5391	1	α_2 = 1.011863
q_3	2.2278777	0.5183	1	α_3 = 0.972888

Figure S9. Calibration of lattice distortion caused by nonlinearity of the STM scanner. a, Atomic resolution image of honeycomb lattice, setpoint: V = -2.0 mV, I = 5.0 nA. **b-d**, Line profiles across the lines marked as 1, 2 and 3 in (**a**). **e**, FFT of (**a**). **f**, Line profiles along q_1, q_2 and q_3 directions marked in (**e**). **g**, Zoom-in of the shaded region in (**f**). **h**, Calibration of lattice constant by the formula $q' = \alpha * q$, where q' represents the calibrated vector, and q represents the measured lattice constants along three directions from STM imaging.

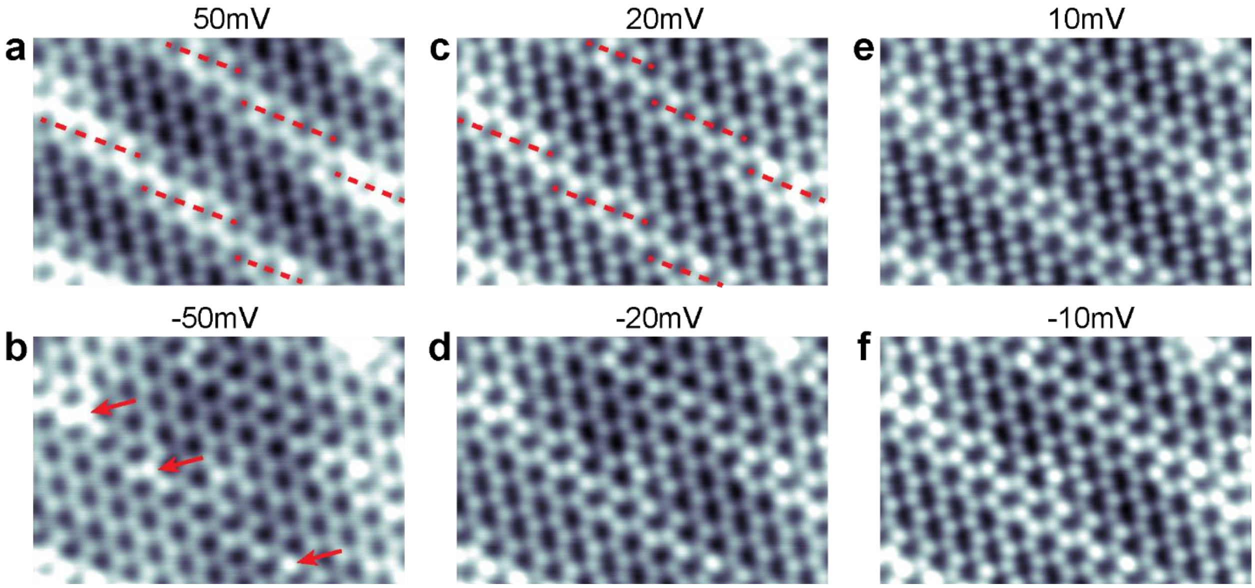

Figure S10. Bias-dependent STM imaging of distorted honeycomb lattice. The red dashed lines in (**a**) and (**b**) indicates the interlaced structure of the stripes. Setpoint: I = 1.0 nA with bias V specified. Size of the image: 4.0 nm × 6.0 nm. The red arrows in (**b**) mark the kink points of the stripes that display a higher contrast under V = -50 mV.

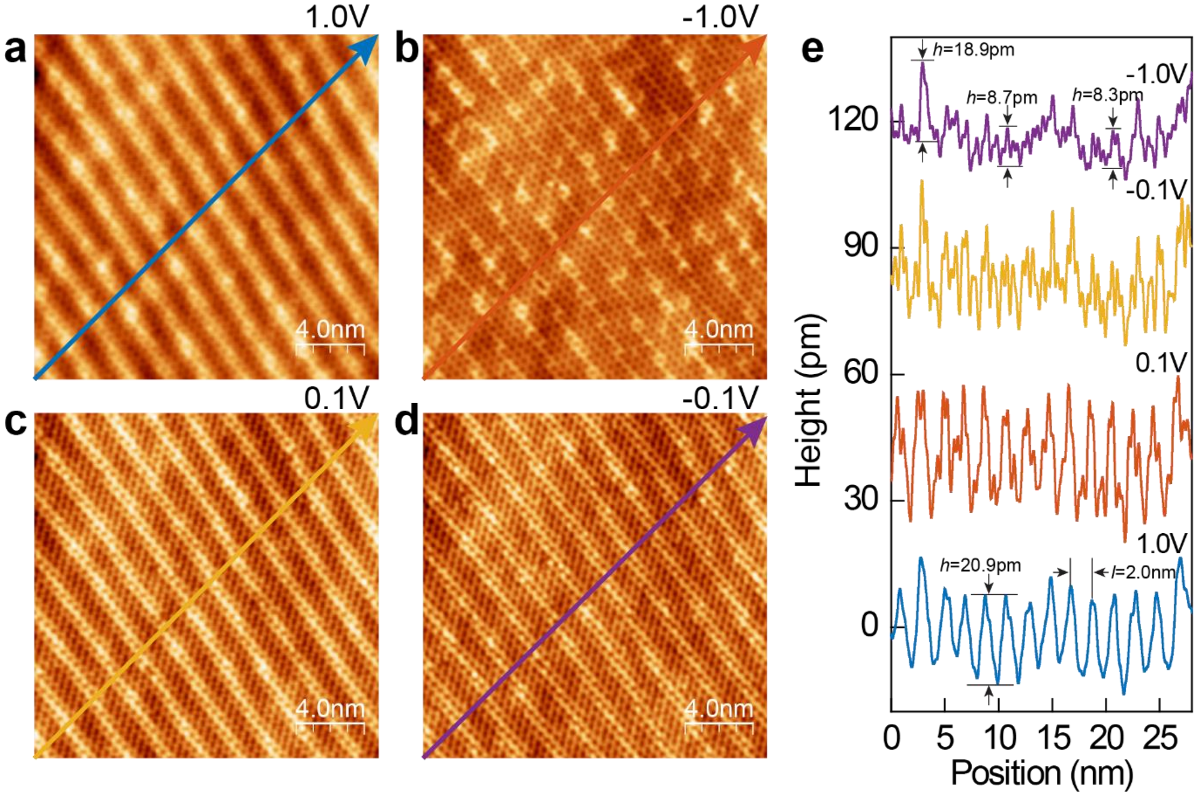

Figure S11. Bias-dependence of stripe modulations. a-**d**, Topographic STM images of stripe modulations under different bias V. Setpoint: I = 5.0 nA with V specified. **e**, Line profile across the topographic image under different bias V with the direction marked by arrows in (**a**-**d**). The high and low contrast under V = 1.0 V contributes to h = 20.9 pm and the periodicity cross the stripes is determined to be l = 2.0 nm.

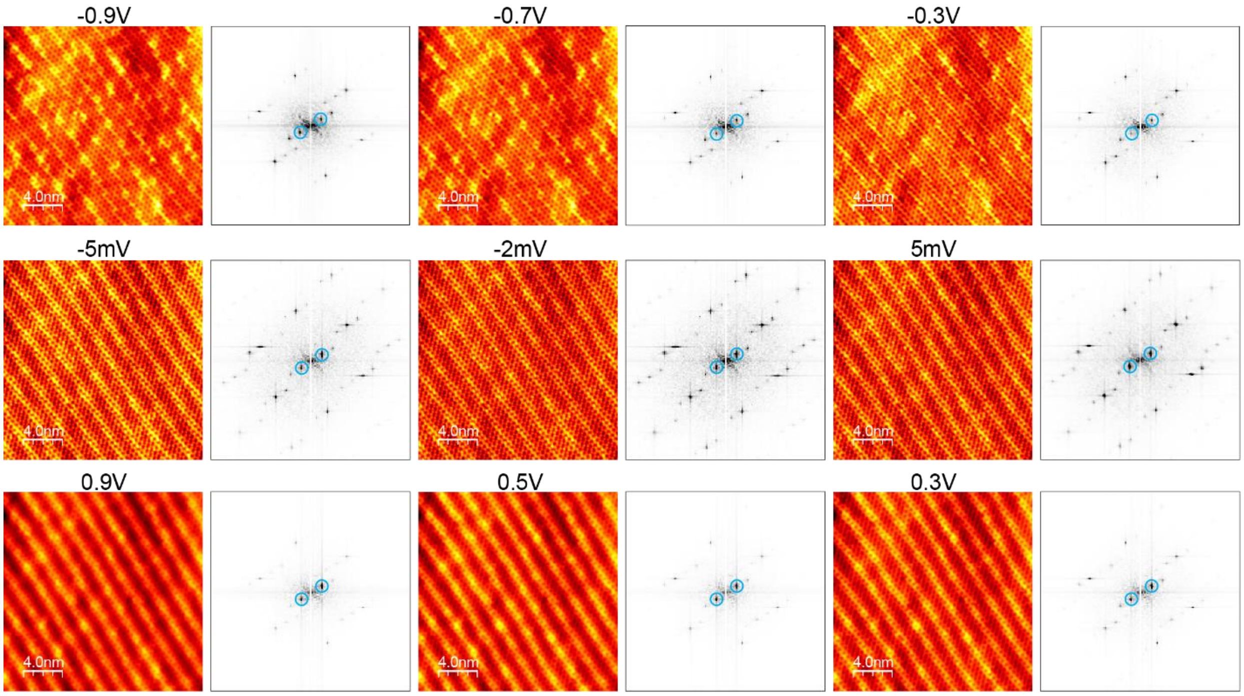

Figure S12. FFT of bias-dependent STM images. The STM images are taken with a tunneling current *I* = 5.0 nA with *V* specified. The diffraction peaks of the stripes are denoted by a pair of cyan circles in FFTs.

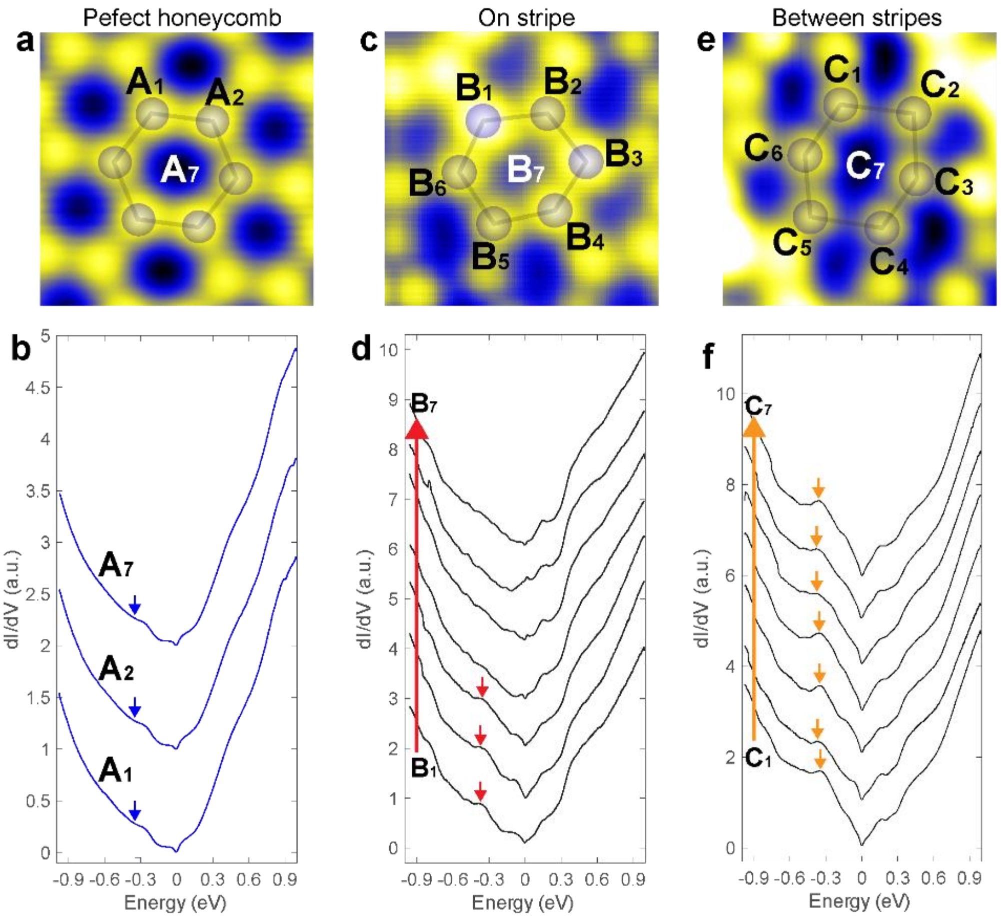

Figure S13. Site-dependent dI/dV spectra within one honeycomb lattice. a, Atomic resolution image showing perfect honeycomb lattice taken on the surface without stripe modulation, setpoint: V = -2.0 mV, I = 5.0 nA. **b**, dI/dV spectra at three typical sites, as labeled A_1, A_2 and A_7 in (**a**). The blue arrows denote the dip feature associated with the Dirac point E_D. **c**, STM image of the slightly deformed honeycomb on the stripe, setpoint: V = 10 mV, I = 1.0 nA. **d**, dI/dV spectra taken at sites labeled in (**c**). **e**, STM image of the strongly deformed honeycomb between the stripes, setpoint: V = 10 mV, I = 1.0 nA. **f**, dI/dV spectra taken at different sites labeled in (**e**). Typical sites of slightly and strongly deformed honeycomb lattice are labeled as B_1 to B_7 and C_1 to C_7, respectively.

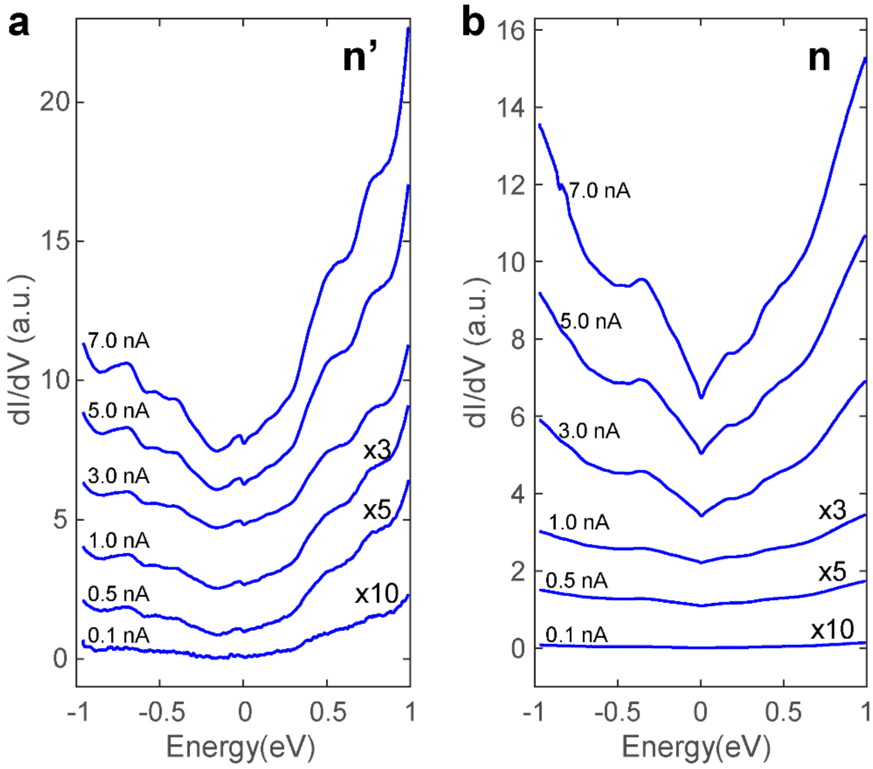

Figure S14. Setpoint-dependent of dI/dV spectra on stripe (n') and between stripes (n). Setpoint: *V* = 1.0 V, with tunneling current *I* specified. Spectra are vertically offset for clarity. The intensity of the dI/dV spectra taken at *I* = 1.0, 0.5 and 0.1 nA are multiplied 3×, 5× and 10×, respectively.

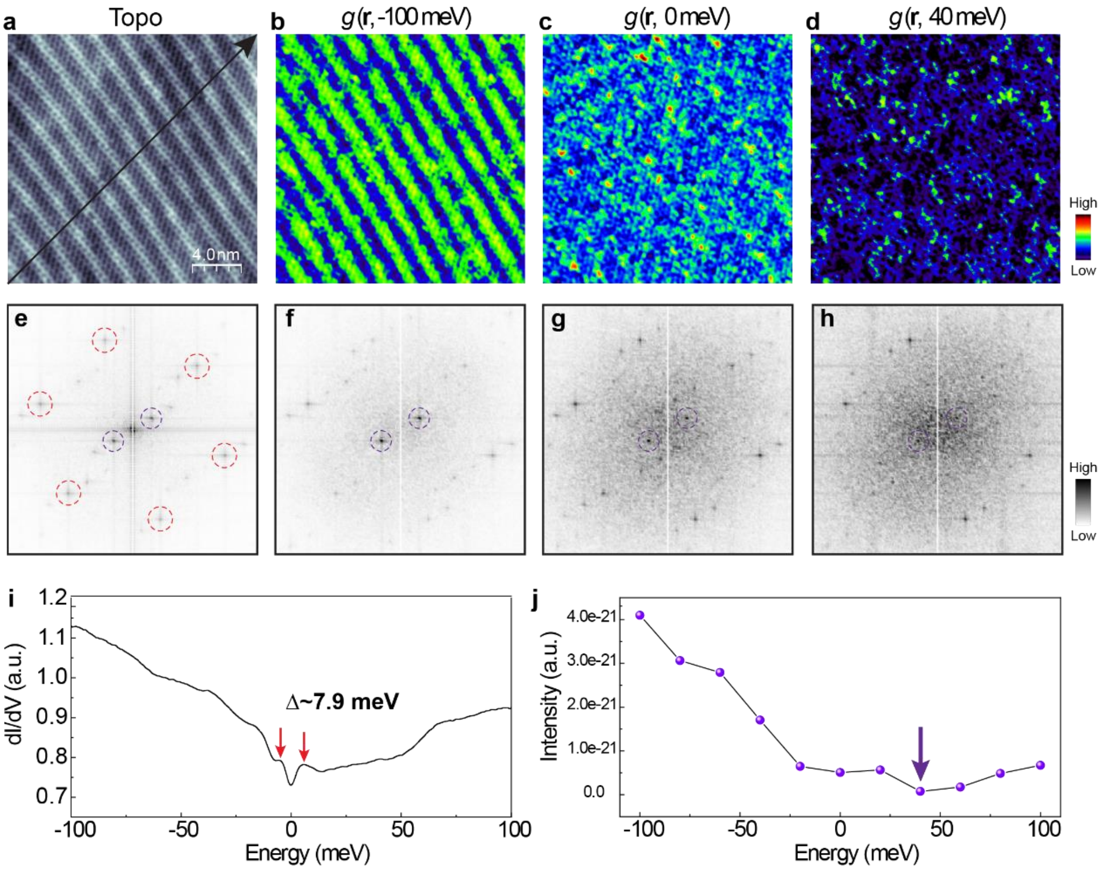

Figure S15. FFT analysis of the stripe modulations. **a**, Topographic STM image showing periodic stripe modulations. Setpoint: V = 100 mV, I = 5.0 nA. **b-d**, dI/dV maps of the region **a**. Setpoint: V = 100 mV, I = 5.0 nA, V_{mod} = 2 meV. **e-h**, FFTs of **a-d**, respectively. The dashed red circles in **e** mark the Bragg lattice. The pair of dashed purple circles in **e-h** mark the stripe feature. **i**, dI/dV maps in the energy range [-100 meV, 100 meV]. The red arrows denote a gap feature $\Delta \sim 7.9$ meV at Fermi level. **j**, The intensity of the diffraction peak for the stripe modulation in FFTs as function of energy, where the minimal is located at 40 meV above the E_F.

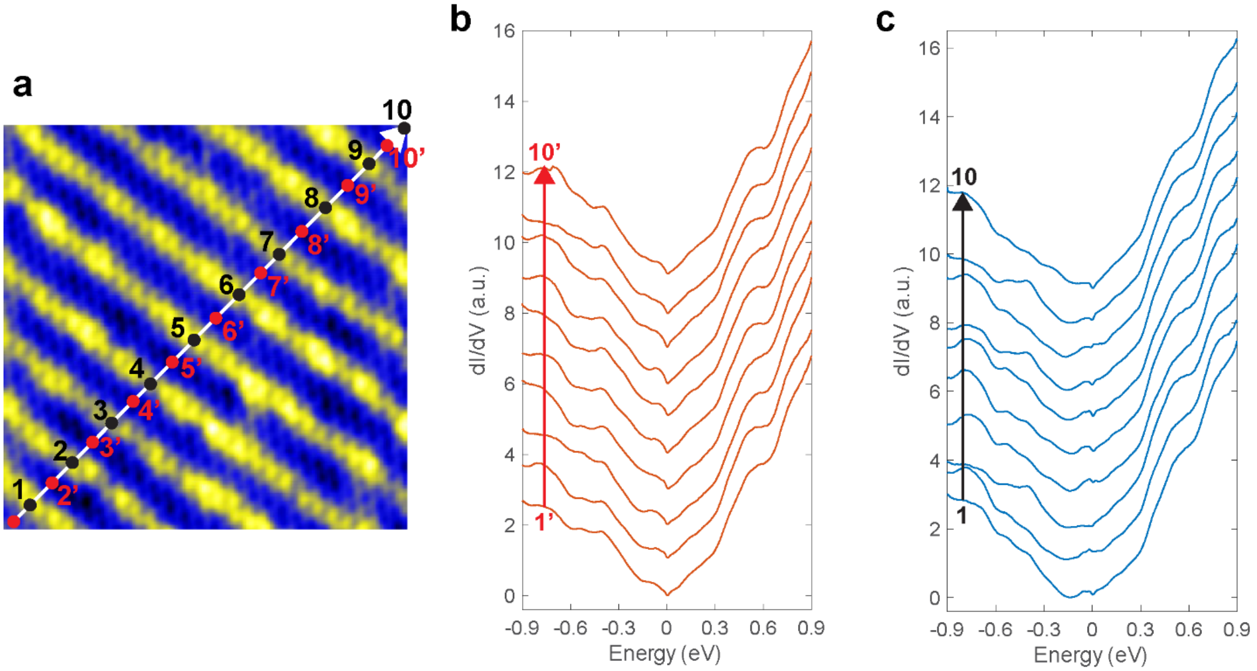

Figure S16. Periodic stripe modulations. **a**, Atomic resolution image of stripe modulations. Setpoint: V = 1.0 V, I = 7.0 nA. 1-10 label sites at the bright pattern (honeycomb lattice) and 1' to 10' label the sites between stripe patterns (deformed honeycomb lattice). **b**, dI/dV spectra at deformed honeycomb lattice, as labeled 1' to 10' in (**a**). **c**, dI/dV spectra at honeycomb lattice, as labeled 1 to 10 in (**a**).

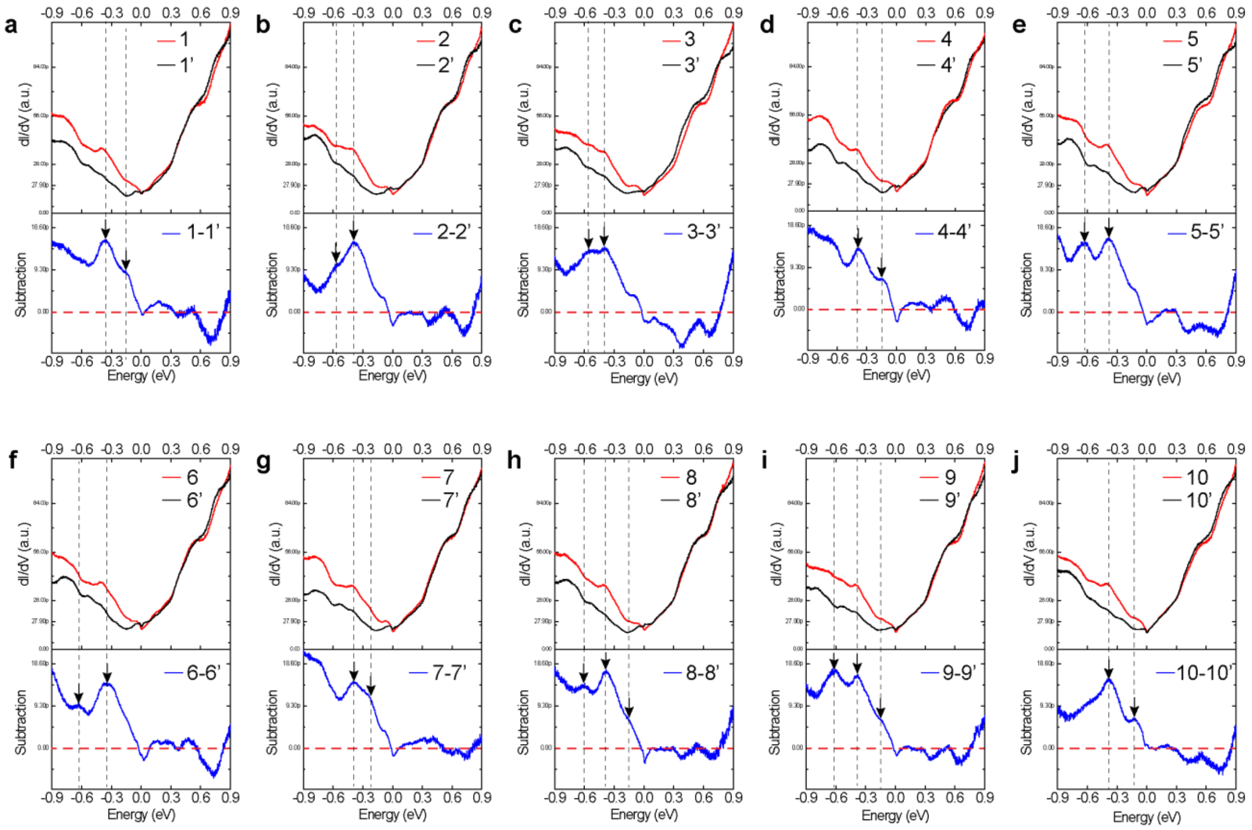

Figure S17. Subtraction of 10 sets of dI/dV spectra to reveal the Landau levels. a-j, dI/dV spectra taken between the stripes (labeled as n) subtracted by that taken on the stripe (labeled by n’). The black arrows and dashed lines mark the Landau levels.

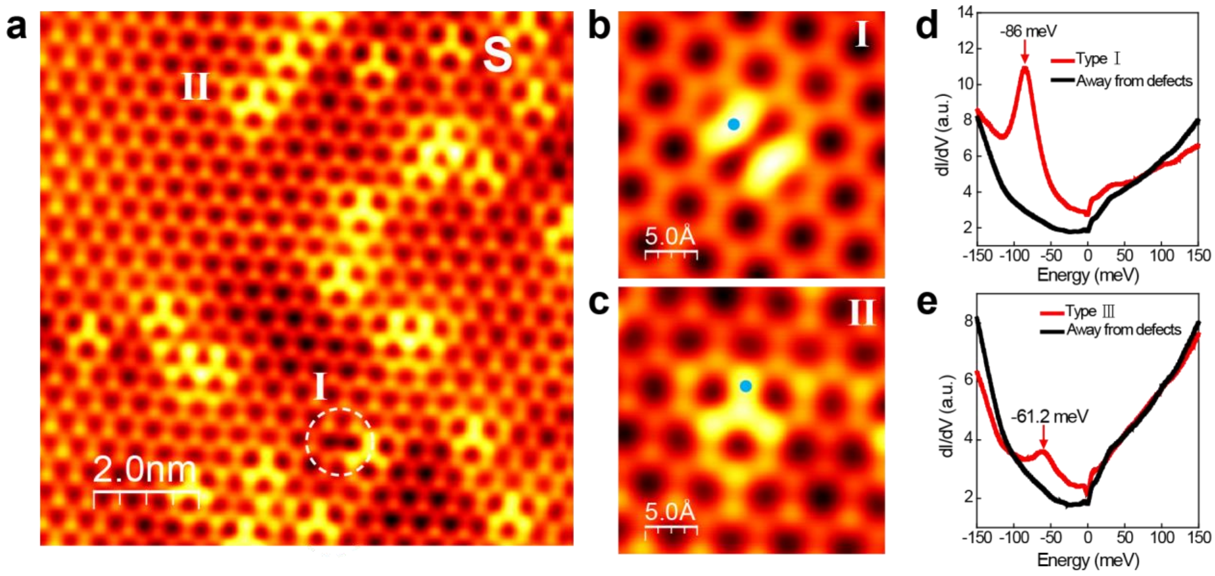

Figure S18. Bound states associated with two types of defects on the Sn-terminated FeSn/STO(111) film. Two types of defects commonly observed on the Sn-terminated surface. **a**, Atomic resolution image of the Sn-termination (S) with a honeycomb lattice with three-fold and two-fold symmetrical defects (dashed white circle). Setpoint: $V = 0.2$ V, $I = 3.0$ nA. **b**, STM imaging showing bound states of the Sn di-vacancy, setpoint: $V = 2.0$ V, $I = 10$ pA. **c**, STM imaging showing bound states of the substitutional defect, setpoint: $V = 2.0$ V, $I = 10$ pA. **d-e**, dI/dV spectra measured at the cyan dots in **b** and **c**, respectively.

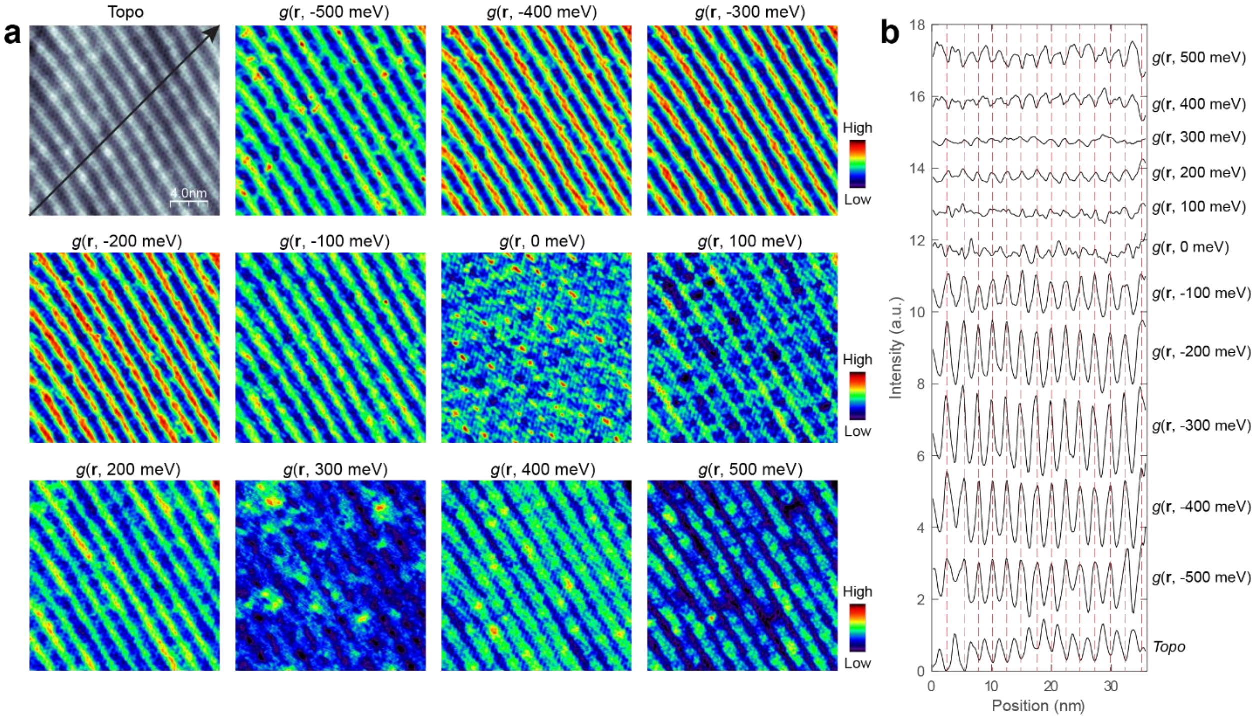

Figure S19. Spatial-resolved dI/dV maps. **a**, Topographic STM image showing periodic stripe modulations. Setpoint: V = 500 mV, I = 5.0 nA. **b**, Line profiles along the black arrow in (**a**) and the energy-dependent dI/dV maps. The dashed red lines mark the valley position from topography. Between the energy ranges [-500 meV, -100 meV] and [100 meV, 300 meV], the valley position in the topography corresponds to higher density of states. At Fermi level $g(\mathbf{r}$, 0 meV), the modulation is almost gone. At the energy window [400 meV, 500 meV], the valley position in the topography corresponds to lower density of states. There is a π-phase shift between the energy ranges of [400 meV, 500 meV] with the other energies. Setpoint: V = 500 mV, I = 5.0 nA, V_{mod} = 10 mV.

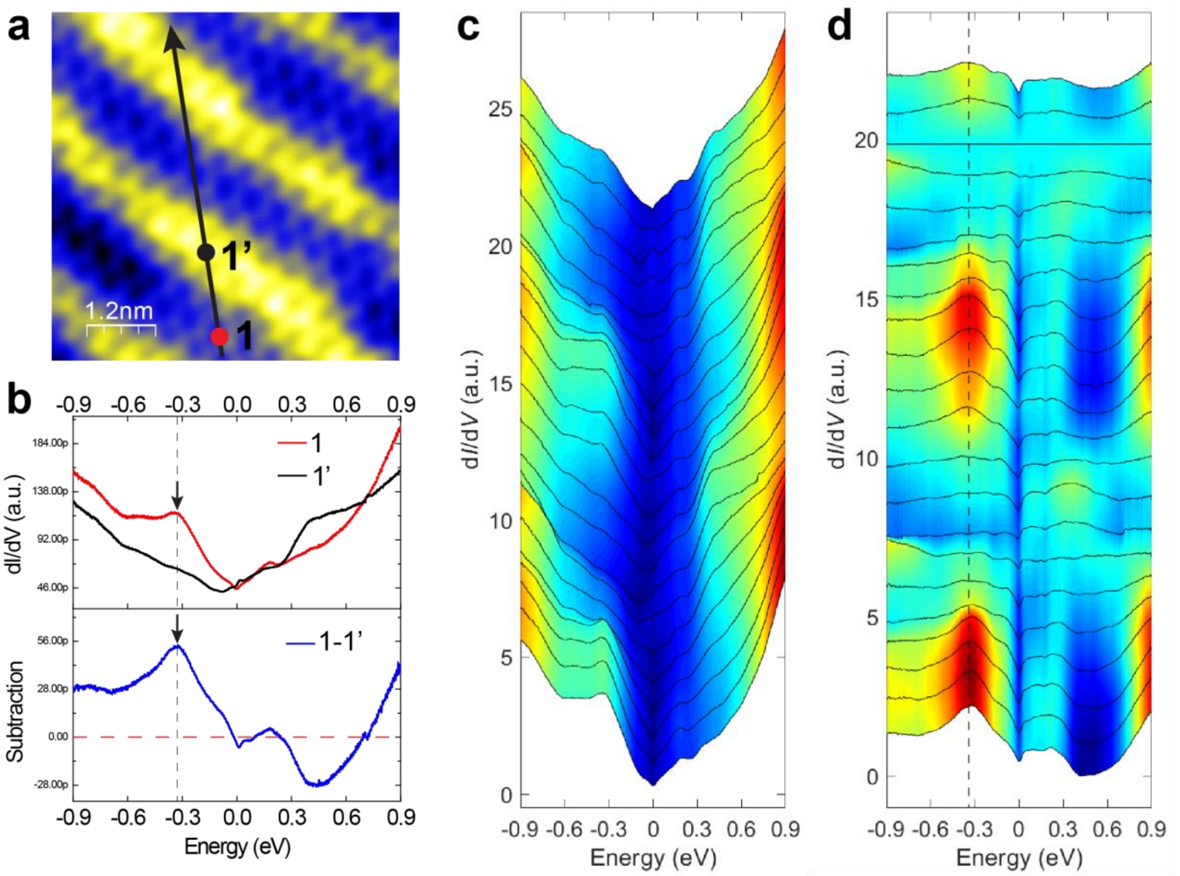

Figure S20. Another example of Landau levels by pseudo-magnetic field. a, Topographic STM image revealing stripes. Setpoint: V = 0.5 V, I = 6.0 nA. **b**, dI/dV spectra taken at the red and black dots (upper panel) and their difference spectrum (lower panel). **c**, A series of dI/dV spectra taken along the black arrow in (**a**). The peak marked by the black arrow is assigned to the 0th landau level. **d**, The dI/dV spectra in (**c**) subtracted by the dI/dV spectrum taken on the stripe. The dashed line clearly reveals the 0th landau level which appears between the stripes and is absent on the stripe.

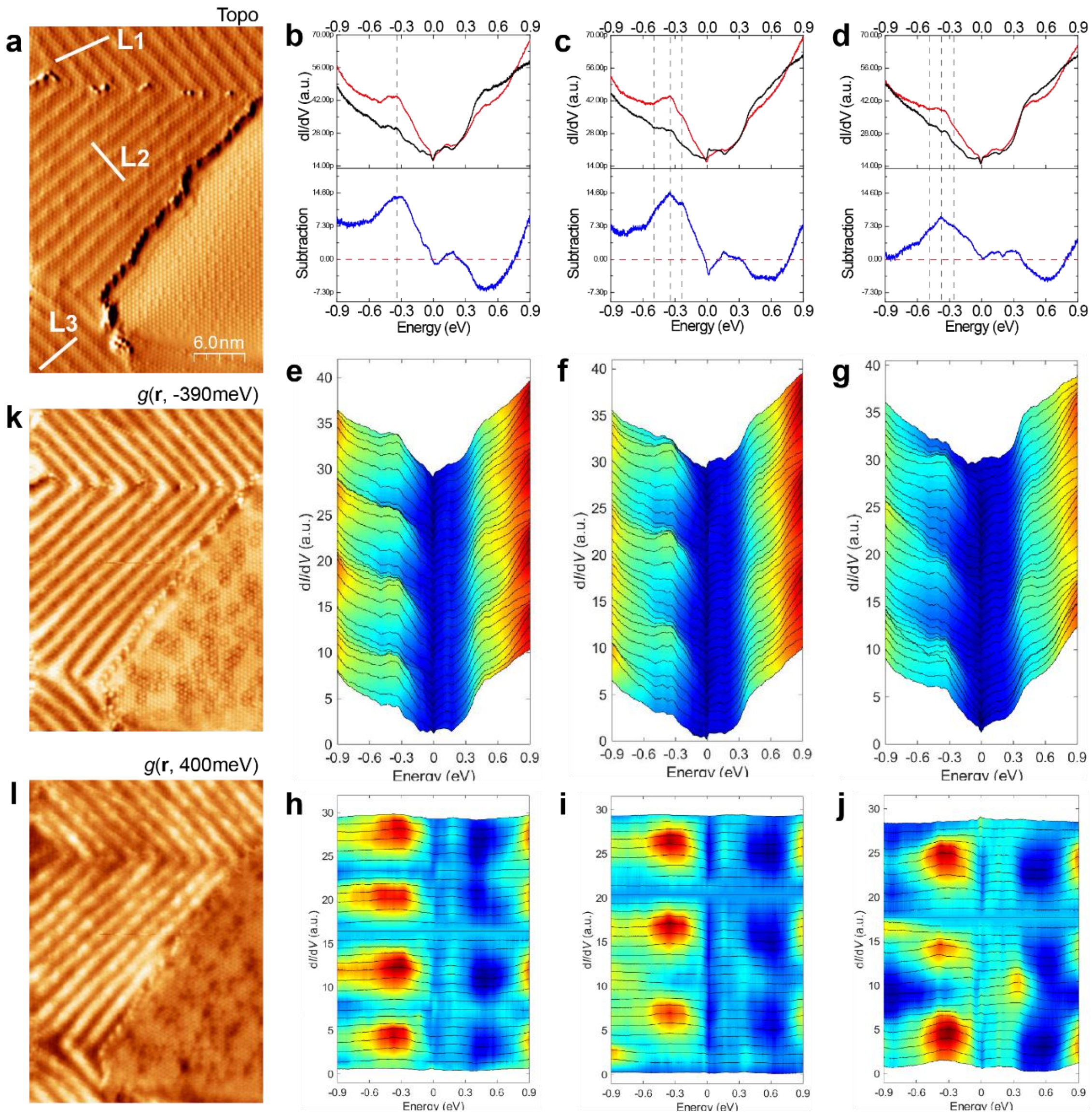

Figure S21. Laudau levels independent on the stripe orientations. a, Topographic STM images revealing the stripes with different orientations. Setpoint: V = 0.2 V, I = 7.0 nA. **b**-**d**, dI/dV spectra taken on the stripes (red curves) and the region between the stripes (black curve) for the line L1, L2 and L3, respectively. **e**-**g**, Line dI/dV spectra taken along the line L1, L2 and L3, respectively. **h**-**j**, A series of dI/dV spectra subtracted by the spectra taken between the stripes. **k**-**l**, dI/dV map at the energy of -390 meV and 400 meV. The contrast of the stripes reverses between the two energies.

REFERENCES

(1) Ilan, R.; Grushin, A. G.; Pikulin, D. I. Pseudo-Electromagnetic Fields in 3D Topological Semimetals. *Nat. Rev. Phys.* **2020**, *2* (1), 29–41. https://doi.org/10.1038/s42254-019-0121-8.
(2) Vozmediano, M. A. H.; Katsnelson, M. I.; Guinea, F. Gauge Fields in Graphene. *Phys. Rep.* **2010**, *496* (4), 109–148. https://doi.org/10.1016/j.physrep.2010.07.003.
(3) Novoselov, K. S.; Geim, A. K.; Morozov, S. V.; Jiang, D.; Katsnelson, M. I.; Grigorieva, I. V.; Dubonos, S. V.; Firsov, A. A. Two-Dimensional Gas of Massless Dirac Fermions in Graphene. *Nature* **2005**, *438* (7065), 197–200. https://doi.org/10.1038/nature04233.
(4) Zhang, Y.; Tan, Y.-W.; Stormer, H. L.; Kim, P. Experimental Observation of the Quantum Hall Effect and Berry's Phase in Graphene. *Nature* **2005**, *438* (7065), 201–204. https://doi.org/10.1038/nature04235.
(5) Xu, Y.; Yan, B.; Zhang, H.-J.; Wang, J.; Xu, G.; Tang, P.; Duan, W.; Zhang, S.-C. Large-Gap Quantum Spin Hall Insulators in Tin Films. *Phys. Rev. Lett.* **2013**, *111* (13), 136804. https://doi.org/10.1103/PhysRevLett.111.136804.
(6) Deng, J.; Xia, B.; Ma, X.; Chen, H.; Shan, H.; Zhai, X.; Li, B.; Zhao, A.; Xu, Y.; Duan, W.; Zhang, S.-C.; Wang, B.; Hou, J. G. Epitaxial Growth of Ultraflat Stanene with Topological Band Inversion. *Nat. Mater.* **2018**, 1. https://doi.org/10.1038/s41563-018-0203-5.
(7) Gou, J.; Kong, L.; Li, H.; Zhong, Q.; Li, W.; Cheng, P.; Chen, L.; Wu, K. Strain-Induced Band Engineering in Monolayer Stanene on Sb(111). *Phys. Rev. Mater.* **2017**, *1* (5), 054004. https://doi.org/10.1103/PhysRevMaterials.1.054004.
(8) Liu, T. Strain-Induced Pseudomagnetic Field and Quantum Oscillations in Kagome Crystals. *Phys. Rev. B* **2020**, *102* (4), 045151. https://doi.org/10.1103/PhysRevB.102.045151.
